# Fractal basins as a mechanism for the nimble brain

**DOI:** 10.1038/s41598-023-45664-5

**Published:** 2023-11-27

**Authors:** Erik Bollt, Jeremie Fish, Anil Kumar, Edmilson Roque dos Santos, Paul J. Laurienti

**Affiliations:** 1 Department of Electrical and Computer Engineering, Clarkson University, 8 Clarkson Ave., Potsdam, NY 13699 USA; 2 Clarkson Center for Complex Systems Science, Clarkson University, 8 Clarkson Ave., Potsdam, NY 13699 USA; 3 Instituto de Ciências Matemáticas e Computação, Universidade de São Paulo, Av. Trab. São Carlense, 400, São Carlos, SP 13566-590 Brazil; 4 Department of Radiology, Wake Forest University School of Medicine, 475 Vine Street, Winston-Salem, NC 27101 USA

**Keywords:** Applied mathematics, Complex networks, Nonlinear phenomena, Dynamical systems

## Abstract

An interesting feature of the brain is its ability to respond to disparate sensory signals from the environment in unique ways depending on the environmental context or current brain state. In dynamical systems, this is an example of multi-stability, the ability to switch between multiple stable states corresponding to specific patterns of brain activity/connectivity. In this article, we describe chimera states, which are patterns consisting of mixed synchrony and incoherence, in a brain-inspired dynamical systems model composed of a network with weak individual interactions and chaotic/periodic local dynamics. We illustrate the mechanism using synthetic time series interacting on a realistic anatomical brain network derived from human diffusion tensor imaging. We introduce the so-called vector pattern state (VPS) as an efficient way of identifying chimera states and mapping basin structures. Clustering similar VPSs for different initial conditions, we show that coexisting attractors of such states reveal intricately “mingled” fractal basin boundaries that are immediately reachable. This could explain the nimble brain’s ability to rapidly switch patterns between coexisting attractors.

## Introduction

It is known that the complex dynamics of the brain exhibits numerous spatiotemporal patterns associated with its many capable responses to a given stimulus, as seen in various imaging techniques. Yet, there has not been a good theory to explain how the system is able to switch among these patterns. Rapidly changing patterns of active brain regions, each containing different types of interconnected neurons that have continuously changing electrochemical properties and environments, only begins to touch on the complexity of a full-scale brain model. This challenge is often countered by course-graining the system to reduce the dimensionality and simplify the model. For instance, instead of analyzing the brain at the neuronal level, even the observational scale of tens of thousands of voxels containing blood oxygenation level dependent (BOLD^1^) signals from functional magnetic resonance images (fMRI) are down sampled to many fewer anatomical or functional brain regions so that functional brain networks of smaller sizes can be analyzed^2,3^.

Experiments using fMRI and other imaging technologies reveal that the brain exhibits a rich variety of activity patterns. While it is generally accepted that certain brain regions are more, or less, active when specific tasks are performed or certain sensory systems such as vision, hearing, or touch are stimulated, it is the global activity patterns that are particularly of interest to us here. An active brain region also implies active neurons, which share information with other neurons and other brain regions. They transmit their signals along axonal pathways via electrical events called action potentials and communicate with other neurons through diverse electrical and chemical synapses^4^. Neural transmission, the process of sharing information along constrained neuroanatomic pathways, can result in neurons exhibiting synchronous large-scale firing patterns, for instance, the collective firing of neurons generating cortical oscillations^5^. In order to understand how the brain processes environmental cues to generate our experiences, thoughts, and/or emotions it is essential that we better understand these ever-changing, i.e. dynamical patterns of synchronous brain activity^5^.

Brain activity can be described mathematically as a complex networked dynamical system which exhibits a key property of multi-stability between numerous states, each associated with different patterns of synchronous activity. The burgeoning field of network neuroscience has used functional brain connectivity^6^ to identify regions of synchronous brain activity, typically assessed using correlations, to show that various patterns of synchrony are associated with distinct cognitive processes^7–9^ or brain disorders^10,11^. Epilepsy, for example, might be understood as a neurological disease of excess synchrony^12^. Most of the time the brain exhibits patchy or partial synchrony, which is a state in which a subset of nodes (or brain regions) synchronizes while activity in other nodes is incoherent^13^. This state of partial synchrony is often referred to as a chimera state, including cluster synchronization^14–16^. We use the term chimera state broadly to describe the presence of coexisting synchronous and asynchronous (meaning disordered) patterns, and saving ourselves the issue of modifiers to allow for various kinds of synchrony in the definition, see details in the SI (Sec. 2). Thus, we consider chimera states as an activity pattern where some subset of the system is synchronous and the rest may be incoherent^17^.

Chimera states have been observed in brain networks at various scales, from small to moderate size neural networks composed of spiking neurons^17^ to brain networks from C. elegans and cats^18,19^. More recently, researchers have extended their investigations to analyze large-scale functional patterns of simulated brain activity using various oscillator models interacting on DTI structural brain networks^20–22^. Spatiotemporal activity patterns over different brain regions fluctuate over time during resting state, so describing brain dynamics in terms of chimera states holds promise, particularly concerning the multistability and metastability of brain activity patterns^23,24^. The key feature of the litany of potential chimera states is that, in a healthy brain, the different organized and disorganized activity patterns coexist with the potential for rapid switching between various states in response to stimuli.

*Mechanism for the nimble brain.* It has been previously observed that the brain is capable of relatively fast task switching and this has been suggested, with both experimental and numerical support^25–30^ to be related to the stability of the basins of attraction involved. Yet, the dynamical mechanisms that underpins the ability of the brain to perform such switching in a rapid manner remain unknown. In particular, why does the basin of attraction of a particular task appear to be quite stable when it is being performed, while simultaneously allowing for ease of switching between tasks? In this work, we propose a potential mechanism for the agile switching between brain activity patterns/states, a process that supports the nimble brain. Using a perspective of dynamical systems, the nimble brain is explained by a complex basin of attraction for each chimera state with multiple states highly intermingled into a fractal basin boundary. Fractal basin boundaries generally involve a large uncertainty in the final state of a multi-stable system^31^. That is, which initial conditions will lead to a particular final state depends on the detailed intricacies of closely packed and intermingled sets associated with disparate basins of attraction^31–35^. In particular, there is an apparently rich “intermingling” of these boundaries, as the present phenomenon of what is called riddled basins^36–38^, that we present in the results. This offers a potential mechanism for agile switching between disparate but complex dynamical patterns, i.e. nimble brain activity, because small changes in current state caused by environmental stimuli would be enough to switch between distinct stable brain states.

An accurate model for capturing the dynamics of the whole-brain has been elusive^24^ and even if such a model existed, it would be premature to use such a complex, high-dimensional system to map the basin structures investigated here. Hence, we adopt a simplified model of spiking neurons on a structural brain network generated using DTI data from a prior study^39^. Much like prior neuroscience research modeling chimera states^20–22^, we located brain-inspired dynamical models, Hindmarsh-Rose (HR) neurons in our case, at each node in the DTI network. As recent research has demonstrated that when coupled, HR neurons can exhibit chimera states under specific parameter settings^18^. Others have used models such as Wilson–Cowan oscillators^20,22^, FitzHugh-Nagumo neurons^21^, as well as Kuramoto oscilators^22^. Regardless of the chosen neural model, this approach allows us to minimize computational complexity while still providing a mechanism to emulate the essential features of the nimble brain’s behavior. Furthermore, we assess the robustness and general applicability of our findings by testing various individual node dynamics, including Kuramoto oscillators and Hénon maps.

We map regions of stability of chimera states to allow us a better understanding of how these disparate patterns co-exist. To make it possible we introduce a technical innovation called the vector pattern state (VPS) that characterizes generalized synchronous behaviour from multivariate time series, allowing for phase and approximate synchronization. Using the VPS technology we are able to cluster similar states from different initial conditions and uncover the underlying riddled basin structure of our brain model. This observation sheds light on a biologically important assertion: fine-scale topological structure of the basins of coexisting chimera states potentially underlies the ability of our nimble brain to rapidly switch between various spatial synchronization patterns.

## Results

### Neuronal model and brain regions

**Figure 1:**
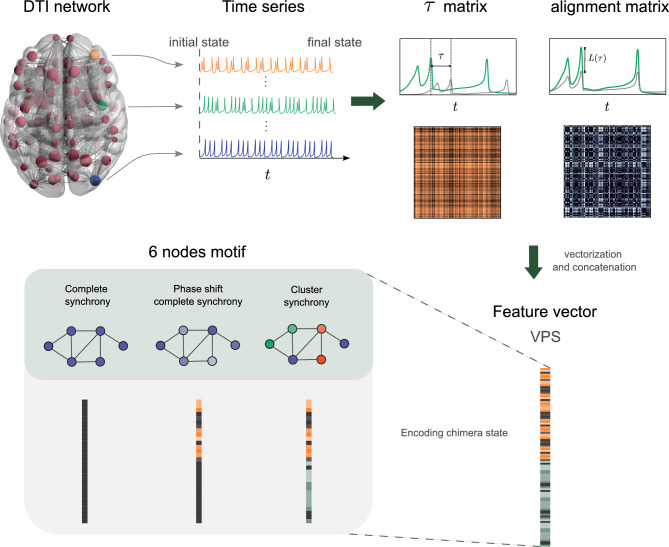
Schematic diagram of the Vector Pattern State construction. (Top) The actual DTI network used in this work mapped to brain space, generated by BrainNet Viewer 1.7 (www.nitrc.org/projects/bnv/)^48^, is shown on the left. Nodes are structural brain regions and the edges are anatomical connections via white matter fiber tracts. The size of each node is scaled by the degree centrality. From some initial state the dynamics of the three individual brain regions are shown as hypothetical time series, reaching a final state. The time shift $\tau$ and alignment between states of all pairs of nodes is recorded at the final state, yielding the $\tau$ and alignment matrix $L(\tau )$. (Bottom) To create a feature vector associated with this final state, we stack and concatenate these matrices into a single vector, defining the vector pattern state (VPS). The VPS encodes patterns of synchrony, with or without phase shift. All states correspond to different VPSs, and are here distinguished in the 6 node network, shown as different colored patterns.

Our phenomenological approach is to leverage the presence of chimera states in neuronal systems as a simplified, yet neurologically relevant, model to illustrate our claims regarding the topological fractal basin boundaries in the brain model dynamics. First, we illustrate the concept of how the brain could switch between disparate pattern states with a semi-synthetic complex coupled system consisting of the well-accepted HR model of spiking neurons, where the coupling structure is a true structural brain network with 83 cortical regions connected by white matter fiber tracts measured using DTI. Figure 1 illustrates the the organization of this network in brain space.

A general model of coupled identical units is given by:

1 $$\begin{aligned} {\dot{{\textbf {x}}}}_i = f({\textbf{x}}_i) + \sigma \sum \limits _{j = 1}^N [A]_{i,j}h({\textbf{x}}_i,{\textbf{x}}_j), \end{aligned}$$

where ${\textbf{x}}_i \in {\mathbb {R}}^d$ is the state vector, $f: {\mathbb {R}}^d \rightarrow {\mathbb {R}}^d$ represents the individual node dynamics, $\sigma \in {\mathbb {R}}^+$ is the coupling strength, *A* is the adjacency matrix describing the coupling structure, and $h: {\mathbb {R}}^d \rightarrow {\mathbb {R}}^d$ is the coupling function. We consider the individual node dynamics given by HR^40,41^ oscillators. For this model, $${\textbf{x}}_i = \begin{bmatrix} x_i, y_i, z_i \end{bmatrix}^T$$, and the individual node dynamics is

2 $$\begin{aligned} f({{\textbf{x}}}_i) = \begin{bmatrix} y_i-ax_i^3+bx_i^2-z_i+I \\ c-dx_i^2-y_i \\ r(s(x_i-x_R)-z_i) \end{bmatrix}. \end{aligned}$$

Above *x* represents the membrane potential, *y* is the rate of transfer of sodium and potassium ions through the fast channels, and *z* is the adaptation current which reduces the spiking rate after a spike has occurred, see SI (Sec. 5.1) for more details about the parameters. We consider diffusive coupling through all variables

3 $$\begin{aligned} h_1(\mathbf {x_i},\mathbf {x_j})= \begin{bmatrix} x_j-x_i \\ y_j -y_i \\ z_j-z_i \end{bmatrix}. \end{aligned}$$

The diffusive coupling mimics electrical interactions between the neurons: a higher difference of ‘+’ and ‘−’ ions between pre-synaptic and post-synaptic neurons causes a proportionally higher flow of these ions through channels. We also consider a more realistic model of the neuronal dynamics, which includes coupling through two terms,

4 $$\begin{aligned} h_2(\mathbf {x_i},\mathbf {x_j})= \begin{bmatrix} 0 \\ y_j-y_i \\ 0 \end{bmatrix}- \alpha (x_i-V_{syn}) \begin{bmatrix} [1+e^{-\lambda (x_j-\theta _{syn})}]^{-1} \\ 0 \\ 0 \end{bmatrix}. \end{aligned}$$

The first coupling term in Eq. (4) describes simple diffusive coupling through the *y*-variables only, while the second represents a “chemical coupling” function. This coupling scenario was presented in^18^ as a more realistic consideration of two types of neuronal connections, one set which interacts through electrical signals and the other does so chemically. An interesting feature of this model was the coexistence of multiple different chimera states, even though the network did not contain any non-trivial automorphism (symmetry) groups. Recently it has been shown that such symmetries are a sufficient^42,43^, but not necessary^44,45^ condition for a graph to support a stable chimera state. This is an important distinction since, in fact, the DTI network that we examine here contains no such non-trivial automorphism group. Indeed, as the number of nodes in a network increases, the lower the likelihood that the network will contain such symmetries^46^.

*Simplification is the first step.* Our model of the brain dynamics incorporates simplifications, where we employ a single-neuron model to represent the dynamics of a node. While more complicated approaches such as the Wilson–Cowan nonlinear oscillator^20,47^ or the neural mass model^24^ could better represent large pools of neurons, the intricacies involved, such as higher-dimensional descriptions and noise, might obscure the essence of our observations. Addressing these challenges in more elaborate models is a task for future research.

### Vector pattern state

At some chosen initial time ($t=0$) the network is in a particular initial state, see Fig. 1. Each node undergoes some dynamics, shown as a time series, and after a transient time, reaches a final state. Out of all time series generated by the network, three are depicted in Fig. 1. Each of the nodes can be classified based on their level of activity by assigning each node a color based on intensity, and nodes with approximately the same level of activity are given the same color.

A chimera state generally describes a scenario amongst *N* coupled dynamical nodes^16,49^ whereby their time variables ${{\textbf{z}}}(t)=({\textbf{x}}_1(t),{{\textbf{x}}}_2(t),\ldots ,{{\textbf{x}}}_N(t))$ (in the notation here, ${{\textbf{x}}}_i(t)\in {{\mathbb {R}}}^3$ denotes one of the coupled HR oscillators; in Eqs. (1)–(2), ${{\textbf{z}}}(t)\in {{\mathbb {R}}}^{3N}$ encompasses the set of all the coupled variables) eventually converge to a state where some of the variables at nodes synchronize, $t>0$, possibly including a phase shift, while others of the variables are incoherent to those, but possibly synchronous amongst themselves. The latter scenario, with the remaining variables being synchronous amongst themselves, is also called cluster synchrony^42,50^.

Traditionally, activity patterns have been identified in terms of the level of synchrony of the overall system^24,51^. However, the system may exhibit synchronous, asynchronous, and partial synchrony, which encompasses chimera states. However, partial synchrony limits a richer characterization of the possible activity patterns. Indeed, for a large system such as the DTI network of $N = 83$, the chimera states can be plausibly quite complex, with exponentially many plausible groupings, and many in fact are feasible. Thus, the characterization of different chimera states requires deciding which variables synchronize in the complex networked system of HR oscillators.

To characterize a chimera state of the 83 brain regions, we quantify the level of synchrony between pairs of nodes in the network. More precisely, after a large time $T_0 > 0$ to allow transients to settle, the time series $x_i(t)$ are compared to $x_j(t-\tau )$ for each *i*, *j* pair, as depicted in Fig. 1. Allowing for phase shift synchrony by a possible shift, we must decide if

5 $$\begin{aligned} L(i,j,\tau )= \lim _{T \rightarrow \infty } \frac{1}{T} \int _{T_0}^{T_0+T} \Vert { {\textbf{x}}}_i(s)- {{\textbf{x}}}_j(s-\tau ) \Vert _2^2 ds, \end{aligned}$$

is small for any phase shift $\tau >0$, which may be decided by minimizing $L(i,j,\tau )$. Here the limit to infinity means large enough integration time, see SI (Sec. 4.1) for practical implementation for finite time series. Since the maximum of the cross-correlation has the property that,

6 $$\begin{aligned} \underset{\tau }{\text{ argmax } }({ {\textbf{x}}}_i\star {\textbf{x}}_j)(\tau )=\underset{\tau }{\text{ argmin } } L(i,j,\tau ), \text{ each } i,j=1,2,\dots ,N, \end{aligned}$$

it is convenient to estimate when variables ${\textbf{x}}_i(t)$ and ${{\textbf{x}}}_j(t)$ settle into a synchronous state by maximization of the discrete cross-correlation,

7 $$\begin{aligned} R_{x_i,x_j}(\tau ) = \sum \limits _{t} x_i(t)x_j(t-\tau ), \end{aligned}$$

in terms of the scalar $x_i$, the first index of each ${\textbf{x}}_i$.

After all pairs are taken into account, we construct the corresponding $\tau$ matrix and the alignment matrix via $L(\tau )$. From these matrices, we create the feature vector, the vectorization and concatenation of the two matrices into a single vector, which we call the vector patterns state (VPS)

8 $$\begin{aligned} e_l=(\tau ^*_{1,2},\tau ^*_{1,3},\dots ,\tau ^*_{N-1,N},\beta L(1,2,\tau ^*_{1,2}),\beta L(1,3,\tau ^*_{1,3}), \dots ,\beta L(N-1,N,\tau ^*_{N-1,N})), \end{aligned}$$

where the parameter $\beta \ge 0$ scales the importance of contrasting the optimal phase shift $\tau ^*_{i,j}$ for comparison of the coupled components, and that best matched difference between components $L(i,j,\tau ^*_{i,j})$. Whether complete synchrony, cluster synchrony, or chimera, with or without phase shift, all patterns are encoded via the VPS, as illustrated in Fig. 1.

### Fractal basin structure supports the nimble brain

Basin of attraction is defined as the set of all the initial conditions in the phase space whose trajectories eventually fall into a particular attracting state. In our case, different initial conditions may lead to the same final state (and are assigned to the same color when visualized) according to the VPS. It is the pairing of the initial state with the final state which we are interested in. This represents the structure of the basin of attraction to various final states.

Recently, there has been significant research into unraveling the basin structure of attractors in high-dimensional systems^52–55^. Typical questions about basin structure have centered around the size and shape of these basins, both quite challenging in our specific case. We are dealing with a system comprising 83 nodes, each associated with a three-dimensional dynamical model, with a phase space that is $3\times 83=249$ dimensional. In contrast to many current studies that rely on characterizing states based on identical synchronization, our focus is on achieving approximate synchrony. We find this approach more versatile and applicable to a broader range of neuroscience questions where identical synchrony is unlikely. Hence, mapping the basin of attraction structure of the various chimera states based on approximate synchrony becomes a problem of associating many long-time patterns from distinct initial conditions, and so this requires a way to match similar signals corresponding to occurrences of disparate chimera states. The full basin structure is too complex to visualize, hindering any chance to uncover its structure, and consequently, the mechanism of the nimble brain. To this end, we use the introduced VPS to solve this mapping problem.

We wish to partition a randomly selected “slice” of the phase space into those regions with similar asymptotic behavior, by observing a sample of *M* initial conditions which we index by *l*, ${\mathcal Z}=\{{{\textbf{z}}}_l(0)\}_{l=1}^M$. To this end, we wish to decide the synchrony pattern of any one ${{\textbf{z}}}_l(0)$, by comparing the long time state of component time series according to Eq. (5) at optimally matched phase shift, according to Eq. (7). With the VPS, we can now assert that two initial conditions ${{\textbf{z}}}_{k_1}(0)$ and ${{\textbf{z}}}_{k_2}(0)$ yield asymptotically similar complex synchrony patterns only if their VPS are relatively close, i.e. $\Vert e_{k_1}-e_{k_2}\Vert _2$ is small.

**Figure 2:**
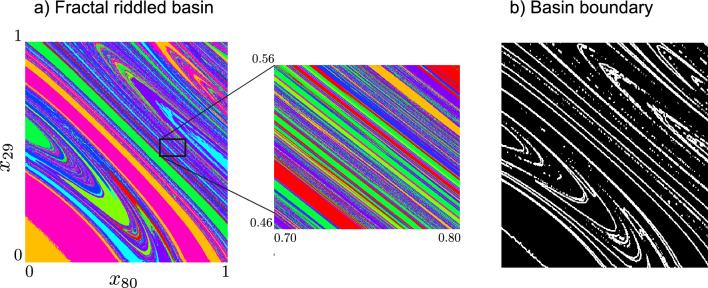
Fractal riddled basin of the full featured HR oscillator model on the DTI network. (**a**) An arbitrary plane “slicing” through the full high dimensional space was selected on which initial conditions are sampled uniformly. Here the *x* component of the 29th oscillator and the *x*-component of the 80th oscillator, at $t = 0$ define the plane. In this basin, the initial conditions associated with different chimera are each a different color. Note that in a region that appears to alternate between just a few states, actually exhibits a rich structure with many different interleaved states when zoomed in at higher resolution. (**b**) The basin boundary set shown in (**a**). The box counting fractal dimension of the basin boundary in this plane, which is computed $d_B\sim 1.8$, being non-integer indicates a fractal set. We consider full featured HR oscillator model Eqs. (2), (4) with $a=1,b=3,c=1,d=5,s=4,r=0.005,x_R=-1.6,I=3.25,\sigma =0.5,\alpha =0.03, V_{syn} = 2, \theta _{syn} = -0.25$ and $\lambda = 10$. The partition into basin structure associated with distinct dynamical chimera states follows k-means clustering on the VPS structure, Eq. (8), using the cost Eq. (5), inferred with cross-correlation, Eq. (7), using $k=8$, the result of a classic elbow method.

Now the problem of partitioning the phase space into like asymptotic chimera states reduces to a clustering problem of all VPSs relative to the different initial conditions. To this end we apply the k-means method to the set of VPS, $\{e_l\}_{l=1}^M$, to cluster the space into k-regions (colors) and we map the phase space by associating these colors to each corresponding initial condition ${{\textbf{z}}}_l(0)$. Thus the clustering is a partition function, ${{\mathcal {P}}}:{{\mathcal {Z}}}\rightarrow \{1,2,\ldots ,k\}$, as shown in Fig. 2. We describe these as basin plots since in any like colored region, the orbits of the initial conditions map asymptotically to similar patterns. Relevant details concerning the experimental methods are included in the figure caption. As noted above, a key component of our method in determining how to group the final states into their various attractors is clustering. While numerous clustering methods exist, we chose, for reasons of computational complexity, k-means. Thus a general description of the k-means algorithm as a clustering method, and the manner in which we choose how fine to partition the space with the selection of a specific *k* are both presented in the SI (Sec. 4.3).

### Coupled HR oscillators in a DTI network

Even with these simplified dynamical models of the brain, there is still rich complexity that demonstrates interesting phenomena in the basin structure. In Fig. 2 we show that using the coupled HR oscillator model, the basin boundary between the states has a non-integer box counting dimension, and thus fractal basin boundaries. In the parameter regime $a = 1, b = 3,c = 1,d = 5,s = 4,r = 0.005,x_R = -1.6,I = 3.25,\sigma = 0.5,\alpha = 0.03, V_{syn} = 2, \theta _{syn} = -0.25$ and $\lambda = 10$, which is known to contain chimeras^18^, we use the electrical and chemical coupling functions, Eq. (4), where the corresponding adjacency matrices are assumed to be the same, unlike in^18^. Here for the first time, we map the manner in which these states are intricately co-mingled. On an arbitrary plane, in this case, which we selected randomly as a slice of the full phase space restriction for the sake of visualization, a uniform grid of $750 \times 750$ initial conditions is chosen. The various colors label initial conditions associated with differing chimera state states. Furthermore, “zoom” restrictions of the domain are also shown to illustrate the fractal-like structure of the basins of attraction at a finer scale. We validate this assertion by computation, that the basin boundaries projected into the planes shown to have a box counting dimension that is not an integer. The box counting dimension of the boundary sets was found to be fractal in Fig. 2b, where the dimension was estimated to be $d_{\text{ box }}\sim 1.8,$ by the method described in Eq. 11.

The basin structure in Fig. 2 appears to exhibit complexity beyond simple fractal basin boundaries. A riddled basin structure appears, which is the scenario that regions exist where points in the domain of one attractor have the property such that small neighborhoods of nearby points have a nonzero probability of being in the basin of another attractor^36–38^. In practical terms, this means that there are large regions in phase space where it is likely that even small perturbations can send the outcome to regions corresponding to a different state. This has significant implications for the possibility of nimble switching between states, since switching between multiple states that may be co-mingled in the phase space may require only vanishingly small control inputs.

#### Fractal basins are ubiquitous

**Figure 3:**
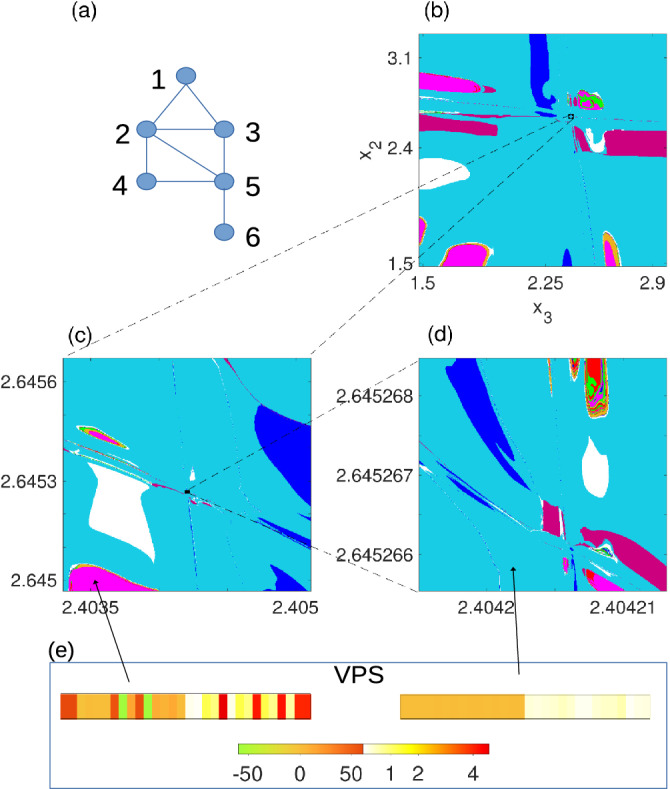
A simplified HR model with diffusive coupling Eqs. (2)–(3) on a small graph illustrates the ubiquity of fractal basin structure of chimera states. (**a**) A network of 6 nodes that does not contain non-trivial symmetries. Nonetheless, there are many stable chimera states (at least on the time scale examined), and the basin structure shown in 8 colors indicates distinct patterns that can be derived by VPS structure, Eq. (8), by the same method as in Fig. 2. (**b**) Fractal basins for HR oscillators on this network when $x_R=-0.5(1+\sqrt{5}), I=3.27, r=0.017, \sigma =0.0004$, and $\beta =1$. All other $x_i, y_i$, and $z_i$ values at $t=0$ are initialized to be $-0.5$. (**c**) and (**d**) are zoomed regions indicated by the black rectangles in (**b**) and (**c**). (**e**) Centroid locations of two of the clusters in $\tau -L$ space, which resembles the approximate form of most of (or all) VPSs inside (see SI (Sec. 2.6) for a detailed view of all $e_l$ vectors inside each cluster).

*HR oscillators coupled in small networks.* To illustrate the generality of our results, we present fractal basins in different networks. Figure 3 displays complex patterns that can be found in the basin of a smaller network of 6 oscillators, as shown in Fig. 3a. We use the electrical coupling scheme with $h_1$ given in Eq. (3), and the parameter values based on earlier research works, see^56,57^. We chose to examine a small synthetic network, which does not have any non-trivial automorphism group, to demonstrate the ability of a coupled HR model to form a basin that has fractal boundaries. In fact, in Fig. 3b, the corresponding estimate is $d_{\text{ box }}\sim 1.27$, where it shows the basin structure grouped into 8 different states using k-means. Figure 3c and d are shown in zoomed (restricted) in regions of Fig. 3b. The structure of the basin is quite complex at all scales examined.

**Figure 4:**
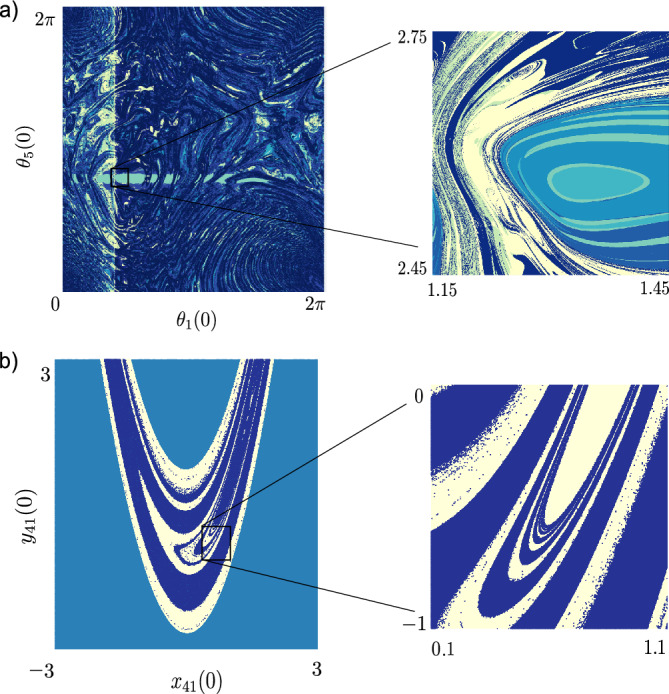
Riddled basins for different networked systems. (**a**) The left panel shows a two-dimensional section of the state space for a system of coupled phase oscillators on a network showing basins of 12 (clustered) distinct states. Right panel Zoomed in from inset of (**a**) showing basins of 7 (clustered) distinct states. To construct the VPS, we use $\beta = 1$ in Eq. (8) and a grid with $1248 \times 1248$ and $624 \times 624$ for left and right panels, respectively, uniformly sampled initial conditions. (**b**) Hénon map dynamics on a DTI network with no non-trivial symmetry. See further details in the SI.

We further explore two more examples of local dynamics and network structure to support the generality of our claims on the nimble brain. In Fig. 4 we illustrate these examples, and thus the ubiquity of complex basin structure between various chimera states.

*Identical Kuramoto oscillators.* We consider the following equations of motion for the identical oscillators

9 $$\begin{aligned} {\dot{\theta }}_{i} = \sigma \sum _{j = 1}^{N} [A]_{i,j} \sin (\theta _j - \theta _i - \alpha ), \quad i = 1, \dots , N, \end{aligned}$$

where $\sigma$ is the overall coupling strength and $\alpha = \pi /2 - {\gamma }$ with ${\gamma } = 0.025$. The adjacency matrix *A* represents a network that does not have full permutation symmetry. To generate this network we initiate two populations of 5 nodes that are globally coupled akin to^58^, and remove uniformly at random one edge from the graph, see details in the SI (Sec. 5.3). Figure 4a shows the complex basin structure that is captured using our VPS.

*Hénon map.* Additionally, we study the network of coupled Hénon maps,

10 $$\begin{aligned} \begin{bmatrix} x_{i}({t+1}) \\ y_{i}({t+1}) \end{bmatrix} = \begin{bmatrix} f_x(x_{i}(t),y_{i}(t)) + \sigma \sum \limits _{j = 1}^N[A]_{i,j}\Big (f_x(x_{j}(t),y_{j}(t)) - f_x(x_{i}(t),y_{i}(t))\Big ) \\ f_y(x_{i}(t),y_{i}(t)) \end{bmatrix} \end{aligned}$$

for $i \in \{1,2,\ldots , N\}$, with $f_x(x,y) = 1-px^2+y, f_y(x,y) = bx$ and $t \in {\mathbb {N}}$, as discussed in^59^. The parameters chosen are $p = 1.44$, $b = 0.164$, $\sigma = 0.8$. The network used is the DTI brain network from Fig. 1. Figure 4b again highlights the generality of the complex structures and also the utility of the VPS technology. Further details of both of these examples are presented in SI (Sec. 5).

## Discussion

The brain has proven to be extremely nimble in its ability to switch between states in response to stimuli, thoughts, and/or decisions. As observed by various imaging techniques, this is associated with rapid switching between patterns of synchronous, chimera, and incoherent states.

*Basin structure of network dynamics*. Several prior works have studied the basin structure of chimera states in networked systems. There have been observations of chimera states with an intermingled basin structure in a special case of a strongly self-coupled cluster network specifically designed to emphasize chimera; see an explanation of critical switching behavior^60^. Authors in^61^ found highly riddled basins in small and highly symmetric all-to-all networks of coupled phase oscillators. Fractal basins of chimeras states were found in small networks of coupled complex maps^62^. In^54^ the authors use a low-dimensional description valid for the infinite size system^63^ to characterize the basin structure of different patterns in a model of two populations of all-to-all coupled Kuramoto oscillators^58^. Likewise and related, in^22^ analyze the same highly symmetric two population network model for chimera was analyzed, but chimera states for a DTI network with coupled Wilson–Cowan oscillator were also illustrated. They define chimera states in terms of a highly approximate synchrony, which is not a general approach such as our VPS that would allow for analysis of basin structure. Similarly, in^19^ chimera premised on approximate synchrony was described for a cat brain connectome data set^64^ describing coupled HR oscillators as coupled through one variable only, but again, no basin structure was found. In^59^, authors use the chaotic Hénon map coupled by again a highly symmetric network, the circulant (ring) stricture, and thus to find fractal basins for chimera premised on identical synchrony.

*Dynamical systems theory is useful to explain the brain.* Dynamical systems theory has been adopted as an approach to gain insights over the brain dynamics across various scales^65–72^. Instead of an empirical or quantitative investigation, e.g. trying to observe attractor-like states^65,73^, most investigations have focused on proposing theoretical dynamical mechanisms^68,70^. For example, dynamical systems theory has contributed to the development of theories of consciousness, by so-called integrated information theory (IIT)^67^, or the description of complex switching phenomenon in biological systems by the concept of chaotic heteroclinicity^69^.

Within a dynamical systems perspective, numerous possible mechanisms exist, necessitating research to pinpoint the one that aligns most closely with empirical data. In this context, we provide numerical evidence of fractal basin boundaries that have non-integer box counting dimension, and riddled basin boundaries.This evidence corroborates a theoretical explanation for resting-state brain dynamics, as investigated in^68^, which shows the promise of this dynamical mechanism. We observe these properties in numerical simulations of multiple different systems of coupled dynamical oscillators, using an experimentally determined human structural brain network as well as small test networks. With this evidence, we have identified a potential mechanism that would allow a nimble brain to switch between various distinct states with only small changes in the system parameters.

From a dynamical systems perspective, we argue that coexisting attractors corresponding to the various chimera states may seemingly suggest that large perturbations would be required to transition from deep in the well of one stable state to another. A brain with such dynamics would be at odds with the idea of a system that can nimbly switch between states. From a neuroscience perspective, it may seem that to transition from one brain state to a distinctly different brain state, one would have to traverse many unique states on a trajectory to the final desired state. We offer an explanation for how to resolve this seeming contradiction in the form of fractal basin boundaries. The fractal basin boundary allows for different stable states to be mixed together closely, creating the opportunity for small perturbations to lead to entirely different stable states, as patterns of chimera.

Thus, the main results of this work are summarized as follows:

1. Our main proposal is that brain activity switching, that is, the nimble brain, is explained by fractal intermingled (riddled) basins. Complex basins of attraction for each chimera state are intrinsically highly intermingled. Thus, significantly different states are nonetheless near each other, in the dynamical variables of the phase space, and so available for nimble control manipulations by internal cognitive processes or external environmental events.
2. Even though the networks in the system have no symmetries, a generalized interpretation of synchrony allows fractal (intermingled) riddled basins, including relatively small model networks.
3. A crucial technology that underpins these above two assertions is based on clustering the VPSs corresponding to chimera states. Here, the k-means of a metric between VPS is a convenient clustering approach. Implementation of the computational task in mapping fractal basins is a key technical innovation that we have developed as background for this new description of the neuronal dynamics of the brain. Our approach can be extended to more complex models of brain dynamics.

Our approach allows the first step to find basin structure of complex high-dimensional systems. Our initial description of such fractal basins necessitated a somewhat simplistic, though biologically inspired, brain model. Now that we have presented this potential mechanism for nimble brain state shifts, experimental neuroscientific studies are needed to empirically validate, or reject, the hypothesis that we have presented. We also envision studies that further investigate the structure of these basins. Promising directions include octopus-like basins for basin structures for chimera states^55^, narrowing down other potential mechanisms for the nimble brain.

## Methods

### Fractal basins: box counting dimension

The assertion of fractal basin boundaries is a matter of considering the approximate boundary set $S_{BL}$, such as the one shown in Fig. 2b, from the basin set in Fig. 2a, shown in cross-section with respect to the variables.

The box counting dimension can be estimated by counting a covering of squares of side length $\epsilon$, and then consideration of this count $N(\epsilon )$ upon refinement by decreasing $\epsilon$. The box counting dimension is defined^74^:

11 $$\begin{aligned} \text{ d}_{\text{ box }}(S_{BL}) = \lim \limits _{\epsilon \rightarrow 0}\frac{\text{ ln }(N(\epsilon ))}{\text{ ln }(1/\epsilon )}, \end{aligned}$$

that is equivalent to the Minkowski-Bouligand dimension. While $S_{BL}$ is simply a slice of the full high-dimensional boundary set, the non-integer result, $\text{ d}_{\text{ box }}(S_{BL})=1.8$, together with the statistically self-similar structure shown, supports the assertion of a fractal set. Likewise, in Fig. 3b, the corresponding estimate is $d_{\text{ box }}\sim 1.27$.

### Supplementary Information

Supplementary Information.

## Data Availability

The network structure used here was derived from diffusion tensor imaging, and parcellated by the Lausanne anatomical atlas into 83 anatomical regions. This structure is publicly available^39^, see the link https://rb.gy/q3o71, from which we selected “Subject 1” as used in^75^. The visualization of the DTI network is generated by BrainNet Viewer 1.7 (www.nitrc.org/projects/bnv/)^48^.
