# Supplementary material for: Fractal basins as a mechanism for the nimble brain
Source: Sci Rep. 2023 Nov 27;13:20860. doi: 10.1038/s41598-023-45664-5 (PMC10682042; doi:10.1038/s41598-023-45664-5)
Supplement: Supplementary file 1 — Supplementary Information. [file 41598_2023_45664_MOESM1_ESM.pdf]

Fractal Basins as a Mechanism for the Nimble Brain

Erik Bollt, Jeremie Fish, Anil Kumar,
Edmilson Roque dos Santos and Paul Laurienti

October 8, 2023

Contents

1 Networks and Complex Systems **2**
1.1 Networks 2
1.2 Subgraphs 2
1.3 Graph Symmetries 3
1.4 Diffusion Tensor Imaging (DTI) Graphs 3

2 Dynamics On Networks, Synchronization, and Chimera States **4**
2.1 Synchronization 4
2.2 Identical Synchronization 4
2.3 Phase Synchronization 5
2.4 Generalized Synchronization 5
2.5 Approximate Synchronization 6
2.6 Chimera States 6
2.7 Nearly Synchronous States 8

3 Basin Structure and Fractals **9**
3.1 Fractal Dimension 9
3.2 Box Dimension 10
3.3 Fractal Boundaries and Riddled Basins 10

4 Computational Methods **11**
4.1 Vector Pattern States 11
4.2 Mapping the Basin Structure 14
4.3 Clustering and k-Means 16

5 Networks of coupled oscillators **18**
5.1 Coupled HR neurons 18
5.2 Two populations of globally coupled oscillators 19
5.3 Coupled oscillators in networks 21
5.4 VPS for phase oscillators 23
5.5 Coupled Hénon maps 23

Here we expand concepts with further details as they appear in the body of this work. Specifically, network concepts for complex systems, dynamical systems, fractals concepts, and machine learning concepts for neuro-inspired models of the brain.

1 Networks and Complex Systems

1.1 Networks

Networks play an important role in many complex systems, including the brain. A networked representation of the human brain, where different regions are treated as vertices and interactions between them as edges, allows us to explore both the structural and dynamical properties.

In this work, we use the phrase network synonymously with the mathematical conception of a graph $G = (V(G), E(G))$ which is a set of vertices (nodes) $V = \{v_1, v_2, ..., v_N\}$, with cardinality $|V(G)| = N$ and edges (links) $E \subseteq V \times V$ with $|E(G)| = M$. The adjacency matrix A is a matrix representation of the graph with entries

$$[A]_{i,j} = \begin{cases} w_{i,j} \text{ if } (v_i, v_j) \in E \\ 0 \text{ otherwise,} \end{cases} \tag{1}$$

where $w_{i,j}$ is a weight. A simple graph is an unweighted graph with no self-loops or multiple edges. Thus, each entry $[A]_{i,j} \in \{0, 1\}$ and $[A]_{i,j} = 0$ if $i = j$.

1.2 Subgraphs

Informally, a subgraph is a graph that results from the erasure of some of the vertices and edges of the original graph. That is, a graph $H = (V(H), E(H))$ is called a subgraph of a graph G if $V(H) \subseteq V(G)$ and $E(H) \subseteq E(G)$ and $|V(H)| = N' \leq N$, $|E(H)| = M' \leq M$. Now suppose the vertices in G are relabeled so that the corresponding vertices in H have the same label, that is $V(G) = \{v_1, v_2, ...v_N\}$, and $V(H) = \{v_1, v_2, ..., v_{N'}\}$. Then we call

H an induced subgraph of G if $E(H) = (V(H) \times V(H)) \cap E(G)$. In other words, if for every vertex in H, the edges formed between each pair of those vertices in the graph G are also found in H, then H is an induced subgraph. A graph is called a proper subgraph if it is a subgraph of G and it is not identically the same as G, i.e., it has strictly fewer nodes than G. Suppose a graph is partitioned into two proper induced subgraphs $H, K \subset G$ such that $V(H) \cup V(K) = V(G)$ and $V(H) \cap V(K) = \emptyset$; that is, H and K are mutually exclusive induced subgraphs. Then G is called connected if for every such H and K pair in G, $E(H) \cup E(K) \neq E(G)$. That is, if for every pair of proper induced subgraphs satisfying the properties above, there is at least one edge between the pair, then G is connected. Said another way, there is a path from every vertex to every other vertex. If G is not connected, then it is called disconnected.

1.3 Graph Symmetries

Symmetry is an important property of graphs that feature prominently in the topic of dynamics on networks, specifically synchrony, though it is not a necessary condition [42]. By symmetry, we mean an automorphism of a graph. A graph automorphism is a permutation of the vertices that preserves the neighbors of any given vertex, meaning the graph remains "essentially" the same. Stated in terms of the adjacency matrix A of a graph, there is a similarity transformation by a permutation matrix P such that $A = P^T AP$, where P can be constructed from the identity matrix by permuting rows and columns only, also here P^T is the transpose of P. Such an automorphism is called a symmetry of a graph if it exists and the graph is called symmetric. Note that the term symmetric here does not mean a symmetric adjacency matrix, but the phrase is usually only used when there exists such a non-trivial permutation. Also, $P \neq I$, the identity matrix, which otherwise always works.

1.4 Diffusion Tensor Imaging (DTI) Graphs

Diffusion Tensor Imaging (DTI) is a magnetic resonance imaging technique used to find the structural organization of white matter tracts in the human brain. Since its formation invention in 1994, the technique has been widely used in brain research. The DTI data can be processed to estimate the brain fiber tracts using a technique called tractography. For the graph shown in Fig.1 of the main text, which is Subject 1 from [9], a process called deterministic tractography, which is available in a software package [45] called the diffusion toolkit, was performed. The brain was parcellated into 83 regions of

interest (ROIs) and fiber tracts from each were computed. Using the ROIs as individual vertices and fiber tracts between ROIs as edges, a weighted structural brain network was generated. The DTI network is then converted to a simple graph as seen in Fig.1 of the main text by changing all non-zero weights to 1 as was done in [13]. When we refer to a DTI network throughout the main text and in the SI, it is this graph that we are referring to.

2 Dynamics On Networks, Synchronization, and Chimera States

2.1 Synchronization

In a coupled dynamical system, synchronization means a '*rhythm*' or '*a kind of sympathy*' between two or more of its dynamical entities. The simultaneous firing of neurons in the human brain, synchronized flashing of light by fireflies, and synchronized clapping by an audience are some of the popular examples [34]. Synchronization arises due to interactions between dynamical elements: while the individual units try to move according to their own set of rules, attractive interactions or coupling with others try to bring harmony between them, which in turn leads to synchronization. Below, different forms of synchronization are discussed, each of which serves a role in our analysis of patterns in the brain, as deduced by our Vector Pattern States (VPS) to distinguish basins.

2.2 Identical Synchronization

The most basic and perhaps standard version of synchronization is called "identical synchronization" (IS) or "complete synchronization" (CS). In a complex system of coupled dynamically evolving units, this state occurs when all units asymptotically "beat together", identically.

Coupled elements are each modeled as differential equations with a vector field defined by a function f described on a graph as vertices, with the edges describing communication between the vertices, through a coupling function h. A generic model used for study in such systems in continuous time is written as:

$$\dot{\mathbf{x}}_i = f_i(\mathbf{x}_i) + \sigma \sum_{j=1}^{N} [A]_{i,j} h(\mathbf{x}_i, \mathbf{x}_j). \tag{2}$$

Here, $\dot{\mathbf{x}}_i$ represents the derivative of $\mathbf{x}_i \in \mathbb{R}^d$ with respect to time, the pair $\dot{\mathbf{x}}_i = f_i(\mathbf{x}_i)$ represents the isolated dynamics associated with an individual

vertex, $[A]_{i,j}$ are entries of the adjacency matrix and $h : \mathbb{R}^d \to \mathbb{R}^d$ is the coupling function between a pair of vertices. In the case where we have $f_i = f$ $(\forall i)$ (meaning that the uncoupled oscillators obey identical dynamics) and $h(\mathbf{x}_i, \mathbf{x}_j) = \mathbf{0}$ when $\mathbf{x}_i = \mathbf{x}_j$ a synchronization manifold can be defined as

$$\mathcal{M} = \{(\mathbf{x}_1, \mathbf{x}_2, ..., \mathbf{x}_n) | \mathbf{x}_1 = \mathbf{x}_2 = \cdots = \mathbf{x}_n\}. \quad (3)$$

Any collective state of all units which occurs in $\mathcal{M}$ is called identically synchronous. Generally, states that asymptotically converge to the synchronization manifold are of interest, initial conditions which do so are defined as being in the basin of synchronization. Thus the set,

$$\mathcal{B} = \{(\mathbf{x}_1(0), \mathbf{x}_2(0), ..., \mathbf{x}_n(0)) | (\mathbf{x}_1(t), \mathbf{x}_2(t), ..., \mathbf{x}_n(t)) \in \mathcal{M} \text{ as } t \to \infty\}, \quad (4)$$

of all initial conditions that are asymptotically attracted to the synchronization manifold is known as the synchronization basin.

Stability of identical synchronization

In their seminal work relating to identical synchronization, Pecora and Carroll [33] analyzed the (local) stability of the synchronous state of a network coupled system. Let $\vec{x}(t) = (x_1(t), x_2(t), ..., x_n(t))$, then a synchronous state is called stable if starting with $\vec{x}(t) \in \mathcal{M}$, there exists a $\delta > 0$ and $\vec{x}(t) + \vec{\Delta} = (x_1(t) + \Delta_1, x_2(t) + \Delta_2, ..., x_n(t) + \Delta_n)$, with $\Delta_i \neq \Delta_j$ if $i \neq j$ and $|\Delta_i| < \delta$ such that $\vec{x}(t) + \vec{\Delta} \in \mathcal{B}$. That is the synchronous state is called stable if an infinitesimal perturbation to that state away from the synchronization manifold returns to $\mathcal{M}$ asymptotically. In [33] details of the conditions under which an identical system will have a stable synchronous state are stated.

2.3 Phase Synchronization

Phase synchronization between two signals $x_i(t)$ and $x_j(t)$ is a generalization of identical synchronization. Whereas for identical synchronization, $x_i(t) \to x_j(t)$, as $t \to \infty$, for phase synchronization, a slightly weaker but otherwise similar condition must hold after one of the signals is shifted by a phase. If there exists a phase shift, $\tau > 0$ such that $\|x_i(t) - x_j(t - \tau)\| \to 0$ as $t \to \infty$, then the two signals are defined as phase synchronous. This property is prominent in our analysis of brain patterns.

2.4 Generalized Synchronization

Generalized synchronization is another weakening of the concept of synchrony. Two signals $x_i(t)$ and $x_j(t)$ are defined as generalized synchronous if

$\|x_i(t) - f(x_j(t))\| \to 0$ for some nonidentity function $f(x) \neq x$. In the identity case that $f(x) = x$, then this would simply be a statement of identical synchronization. Usually, the statement is in terms of a smooth function f, and the set of points $\mathcal{D} = (x, f(x))$ on which orbits $(x_i(t), x_j(t))$ are attracted is called the synchronization manifold, and this phrase can be used even in the identical case. It is certainly possible for a system to exhibit phase and generalized synchrony.

2.5 Approximate Synchronization

If two signals $x_i(t)$ and $x_j(t)$ approach a synchronous state, such as identical synchrony, but they do not converge to that state, then the synchronous state is stable but not asymptotically stable. In other words, if for some time $T > 0$, and small $\epsilon > 0$, then $\|x_i(t) - x_j(t)\| < \epsilon$ for all $t > T$, but they do not converge to zero, then the signals are approximately (identically) synchronous.

2.6 Chimera States

A chimera state is defined by the presence of both synchronous and asynchronous behaviors in a complex system. Specifically, if a collection of coupled dynamical systems with corresponding signals, $\{x_1(t), x_2(t), ..., x_n(t)\}$ results in at least one subset of these that synchronizes (in terms of one of the types of synchronization noted above), and a distinct other subgroup that does not synchronize, then this is called a chimera state. Amongst those that synchronize, there may be just one large synchronous cluster, or perhaps several clusters that synchronize but not with each other, this is still called chimera, as long as there is also a subset that is asynchronous.

Chimera states have long fascinated the synchronization community, being first named chimera states (in an identical synchronization context) by Abrams and Strogatz in 2004 [2] though this phenomenon was reported earlier by Kuramoto and Battogtokh [20]. Though originally the term chimera state was reserved for identical oscillators, the literature is now filled with examples in which the term chimera state is used for non-identical oscillators [21, 38, 27, 18, 22] and so we have chosen to use the term in the broadest sense. The definition used here allows states that are sometimes called cluster synchronization or simply a clustered state [19, 28, 46, 6, 7], however, it has been observed that a change in the coupling strength is enough to create a chimera state by isolated desynchronization of all clusters except for one [32]. An example of one such representation of a chimera state can be found in Fig. 1(j).

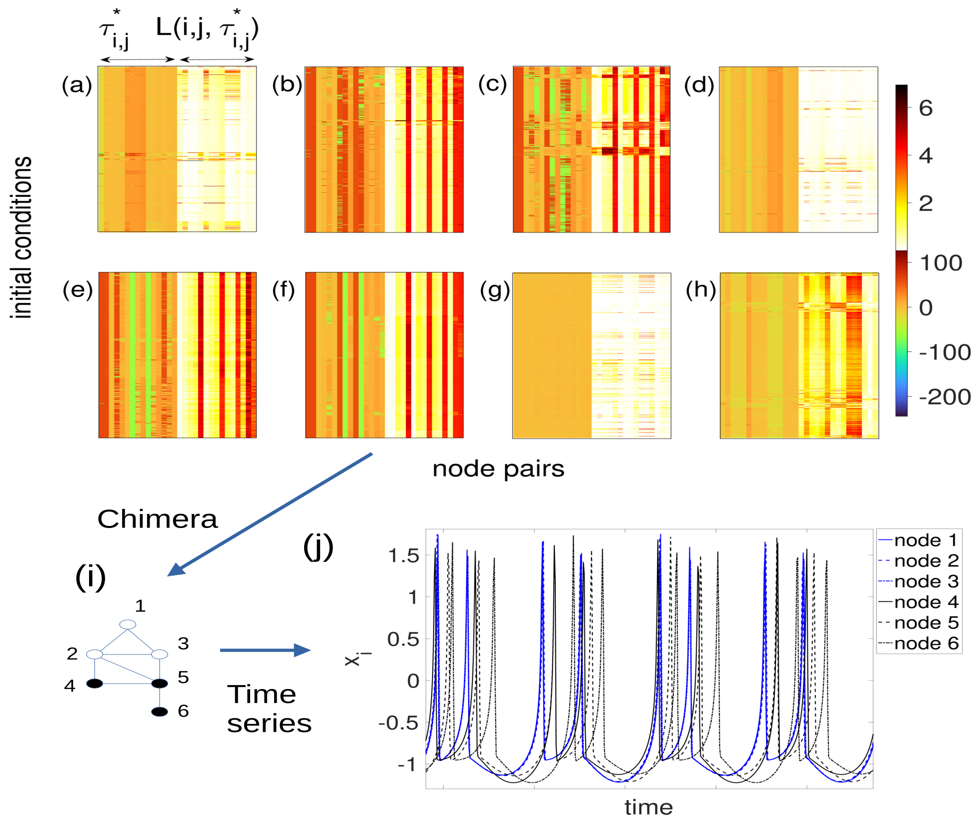

Supplementary Figure 1: All VPSs in Fig. 3 of the main text. (a)-(h) show all e^l vectors (Eq. 12) in each of 8 clusters. The x-axis represents vertex pairs while the y-axis contains initial conditions forming a cluster. The color intensity represents entries of e^l. (i) The color-coded network shows the chimera state in (f). The vertices with a higher degree of synchronization, such that $L(i, j, \tau^*_{i,j}) \approx 0$, are colored white, while the remaining ones are shown in black. (j) shows x_i versus time for this chimera state and is plotted as follows: first, $x_i(t)$ and $x_j(t - \tau^*_{i,j})$ for nodes i and j having the smallest $L(i, j, \tau^*_{i,j})$ value is plotted; thereafter, the phase-shift for any new node k is decided such that $L(i, k, \tau^*_{i,k})$ minimum over all the pre-existing nodes i.

The kind of synchrony present defines the details of the chimera state, whether the synchronous group is identical, generalized, phase synchronous, or approximate. In our synthetic model of HR oscillators on the DTI network as well as smaller models, we observe all of these. The stability of synchrony associated with a chimera state has some dependence on symmetry [32].

2.7 Nearly Synchronous States

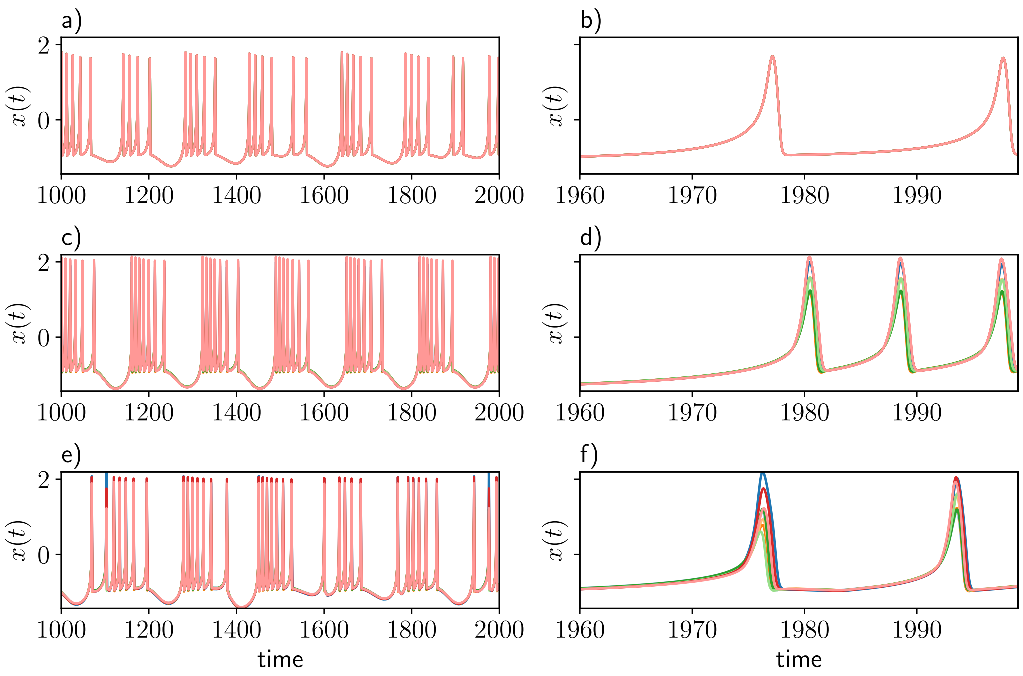

Supplementary Figure 2: Time series of the x-coordinate of the HR neuron model evolving on the DTI network without phase shift. (a) An x-coupled HR neuron model with no chemical coupling (i.e. $\alpha = 0$ and $\sigma = 1.7$), where we only observe identically synchronized oscillators. (b) Zoom in the panel of (a) for better visualization. (c) For chemical coupling $\alpha = 0.03$ (and $\sigma = 1.7$) oscillators still spike at the same time, but have different sized peaks creating a spread in the heights, though they still fully synchronize in phase. (d) Zoom in the panel of (c) for better visualization. (e) The HR model with the same coupling parameters as (c) but with the model parameter a drawn from a normal distribution $a \sim \mathcal{N}(1, 0.1)$. (f) Zoom in the panel of (e) for better visualization. In this case, the oscillators peak in the same general time frame but these spikes have different peaks and different shapes. This represents a more generalized synchronization pattern.

Expanding upon the concept of approximate synchrony, Sec. 2.5, here for the HR model, we synonymously state as nearly synchronous. Consider Fig. 2 (a) and (b) in the model with only electrical coupling, Eq. 2 with dynamics f given by Eq. 18 and coupling function h_1 from Eq. 19, yield identical synchronization as shown in Fig. 2 (a). However, in Fig. 2 (c) we have added a chemical coupling term changing the coupling function h_2 to the one from

Eq. 20, which is the model used by [18] (see Sec. 4). As can be seen, while the neurons still appear to be synchronized in phase, the peaks no longer match. In Fig. 2 (e) we have included both the electrical and chemical coupling terms, and additionally, we have introduced a slight parameter mismatch. The model parameter a, which was previously set to 1 for all oscillators is now drawn from a normal distribution $a \sim \mathcal{N}(1, 0.1)$. This is an example of nearly synchronous states of which is possible to examine the linear stability [44], even in the context of chimera [39].

3 Basin Structure and Fractals

3.1 Fractal Dimension

Examples of fractals are abundant in nature: they exhibit patterns across many scales that are self-similar, in some form. Real world examples, where the self-similarity is approximate and fine details do not continue indefinitely, include the coastlines of Britain (fractal dimension ≈ 1.21) and Norway (fractal dimension ≈ 1.52) [12]. Newton's fractals, Von-Koch curves, Cantor sets, Sierpinski's triangle, and the Koch Snowflake [12] are examples of mathematical fractals that continue indefinitely. Fractals can be understood as structures with a roughness that can be represented by a real number, called their dimension. A definitive property of fractals is the concept of non-integer dimensionality [12], as this fractional dimension motivates the very naming of sets as "fractals". Whereas fractals are all defined on the scaling of data density relative to an exponent describing the dimensionality, manifold dimensionality describes the integer number of coordinates necessary to uniquely parameterize a point. For instance, a straight line is 1 dimensional, a plane surface is 2 dimensional, and a cube is 3 dimensional. Fractal dimensionality is generally a description of the scaling behavior of a set with respect to decreasing window perspective; a non-integer fractal. It is a concept that applies to smooth as well as "rough" sets. From this perspective, the coastline of Norway is rougher than Britain, and correspondingly, its dimension is also farther from nearby integer numbers 1 and 2 [23, 5, 4, 40].

The fractal dimension is formally defined by the Hausdorff dimension, and various popular methods serve as useful estimates. For example, the similarity dimension, the correlation dimension, and the box dimension are each useful data-driven estimators of the fractal dimension with certain underlying assumptions on the data. For practical computational reasons, here we use the box dimension method to calculate the fractal dimension [35, 15, 37].

3.2 Box Dimension

The *box counting dimension* [15] is a popular estimator for fractal dimension. Squares (boxes or hyperboxes in higher dimensions) of side length ϵ are chosen and the number of 'boxes' ($N(\epsilon)$) of this size needed to cover the entire set are counted. The box dimension (d) is understood as the scaling exponent, in the limit as the area (or volume) of the box goes to zero with:

$$d_{\text{box}}(S_{BL}) = \lim_{\epsilon \to 0} \frac{\ln(N(\epsilon))}{\ln(1/\epsilon)}, \tag{5}$$

supposing the limit exists [43]. If the above limit does not exist, one may still find the upper and lower box dimensions of the set. Efficient algorithms for estimating the box dimension exist [37]. Fig. 3(a) shows an example of a boundary set obtained using Eq. 19, while Fig. 3(b) shows the plot of $\ln(\frac{1}{\epsilon})$ vs $\ln(N)$ for this boundary set. The slope of the line of best fit is interpreted as the limit given in Eq. 5, which in this example is equal to 1.27. As expected, the dimension of the boundary set is non-integer. Note that in practice due to finite data set size, as $\epsilon \to 0$, $N(\epsilon)$ saturates after a certain ϵ value is reached, which is due to the fact that $N(\epsilon)$ can not increase further once each point in the set is covered by a unique square.

3.3 Fractal Boundaries and Riddled Basins

Complexity can be found even in the structure of the basins of attraction in multi-stable systems. The feature of interest to this work is the possibility of the concept of a high level of "non-smoothness" of a fractal basin boundary when moving between sets of initial conditions attracted to one basin and those leading to a different one. In particular, we described a scenario that arises for the HR system and some parameters where a particularly highly intermingled fractal basin boundary called riddled basins occurs. We shall describe these scenarios.

In general, a boundary point of a set has the property that every neighborhood of the point, no matter how small, includes some points both in the set and not in the set. The boundary set of a given set is thus the union of all such boundary points. The boundary of an open disc in the plane, for example, is a circle, which clearly is a smooth set. However, in dynamical systems, it is not uncommon that boundary sets of basins of attraction can be fractals. The set of points forming the boundary between the basins of different attractors need not form a smooth curve, and instead, this boundary set may have a fractal dimension [26]. Our proposal herein is that the key feature of complex networked models of the brain with the observed behavior of rapid

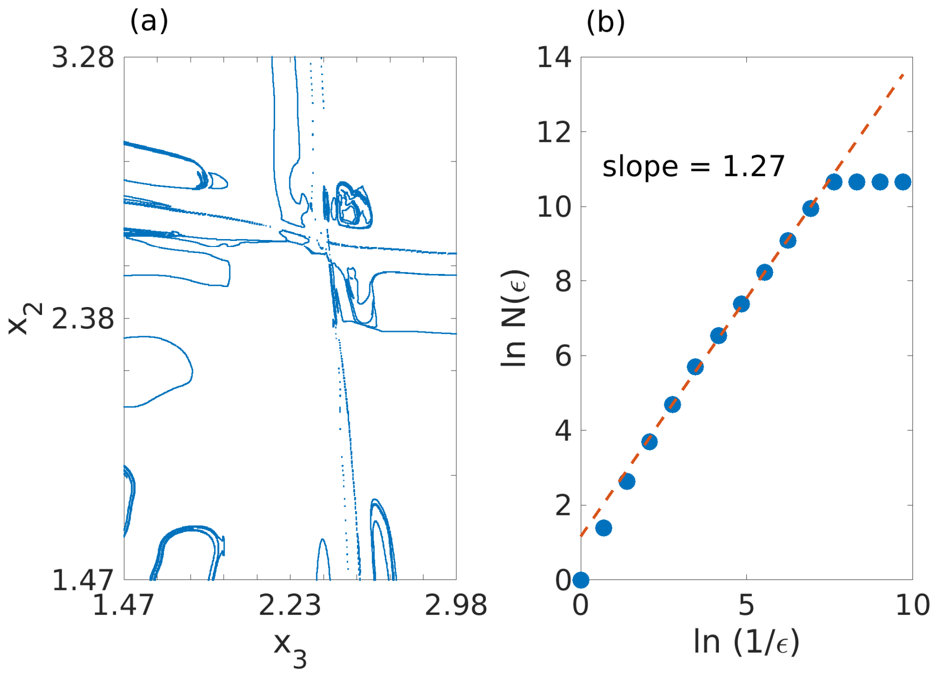

Supplementary Figure 3: (a) The boundary set obtained from Fig.3(b) in the main text. (b) The graph of $\ln(\frac{1}{\epsilon})$ vs $\ln N(\epsilon)$, calculating the dimension of the boundary set in (a). The fitted slope of 1.27 estimates the fractal dimension.

switching between various patterns is the presence of fractal basin boundaries [14, 26]. In particular, there is an apparently rich "intermingling" of these boundaries as the present phenomenon of what is called riddled basins [3, 29, 10, 30]; see Fig. 6. Riddled basins occur in many physical settings, notably in the basin structure, for example, of magnetic potential wells [47], but the fact that we point this out in neurophysiology as shown in Fig. 6, as a crucial mechanism behind the ability to quickly switch focus, the nimble brain theory, is a unique and new interpretation of this now classical concept from dynamical systems.

4 Computational Methods

4.1 Vector Pattern States

Here, we describe in more detail what we have introduced as Vector Pattern States (VPS) that allow comparisons between different initial conditions. The purpose of the VPS is to classify the various fully synchronous, chimera,

and incoherent states that may coexist in the basin. Thus, we associate which components of the complex network are in some form of synchrony, including the weaker approximate synchrony and phase-shifted synchrony as presented in Section 2. This is a crucial technological step for mapping the basins as described in the next section.

In general, synchrony is defined asymptotically (i.e. as $t \to \infty$), which is impractical. Instead for a finite time interval *time series*

$$\mathbf{x}_i(t), \text{ for } t \in [T_0, T_0 + T], i \in \{1, ..., N\}, \tag{6}$$

a similarity measure must be introduced to determine if nodes are 'close enough' to be considered synchronized. Let $I \subset \mathbb{R}$ and $\|\mathbf{u}\|_{L^2(I,M)}$ be the norm given by

$$\|\mathbf{u}\|^2_{L^2(I,M)} = \int_I \|\mathbf{u}(t)\|_2^2 dt, \tag{7}$$

where M is a compact subset of $\mathbb{R}^d$ and $\|\cdot\|$ is the Euclidean norm $\|\mathbf{u}\|_2$ on $\mathbb{R}^d$. We abuse notation and also denote $\|\mathbf{u}\|_{L^2(I,M)}$ by $\|\mathbf{u}\|_2^2$. Also, we denote $u^\tau(t) = u(t - \tau)$ as the function u evaluated at $t - \tau$. Other L^p norms could be used to define the similarity measure, due to the equivalence of L^p norms in finite-dimensional spaces. We will investigate the influence of different norms in future research.

An example of the similarity measure between the trajectories $\mathbf{x}_i(t)$ and $\mathbf{x}_j(t)$ of a pair (i, j) could be an average over $I = [T_0, T_0 + T]$

$$L(i, j) = \frac{1}{T}\|\mathbf{x}_i - \mathbf{x}_j\|_2^2 = \frac{1}{T}\int_{T_0}^{T_0+T} \|\mathbf{x}_i(t) - \mathbf{x}_j(t)\|_2^2 dt. \tag{8}$$

This is fine in the CS case, however as we are interested in a more general synchronization case, the similarity measure should also allow for the possibility of a non-zero time shift τ between the time series. Thus, we consider the following similarity measure

$$L(i, j, \tau) = \frac{1}{T}\|\mathbf{x}_i - \mathbf{x}_j^\tau\|_2^2 = \frac{1}{T}\int_{T_0}^{T_0+T} \|\mathbf{x}_i(t) - \mathbf{x}_j(t - \tau)\|_2^2 dt. \tag{9}$$

The time shift that minimizes Eq.9, $\tau^*_{i,j}$, is of interest in this work since synchronization is viewed from a less restrictive criterion than CS, allowing for time shifts. For real-valued time series ($d = 1$), note that $L(i, j, \tau)$ is related to cross-correlation, in the sense that

$$(x_i \star x_j)_\tau = \int_{T_0}^{T_0+T} x_i(t)x_j(t - \tau)dt \tag{10}$$

is maximized at a particular $\tilde{\tau}$, which happens to be the same $\tau^*_{i,j}$ that minimizes $L(i,j,\tau)$. In other words,

$$\tau^*_{i,j} = \arg\max_{\tau} \; (x_i \star x_j)_\tau = \arg\min_{\tau} \; L(i,j,\tau). \tag{11}$$

Thus, it is possible to find $\tau^*_{i,j}$ using the well-studied method of cross-correlation. In practice, not only is the time interval finite, but the sampling rate is finite as well, allowing the integrals to be replaced by sums and the cross-correlation $R_{x_i,x_j}(\tau)$ can be found using the `xcorr` function in MATLAB. Since we define $\tau^*_{i,j} = \arg\max_{\tau} \; R_{x_i,x_j}(\tau)$, when $T - |\tau^*_{i,j}| > 0$, we calculate $L(i,j,\tau^*_{i,j})$ using the formula

$$L(i,j,\tau^*_{i,j}) = \frac{1}{T - |\tau^*_{i,j}|} \sum_{t=T_0+|\tau^*_{i,j}|}^{T_0+T} \|\mathbf{x}_i(t) - \mathbf{x}_j(t-\tau^*_{i,j})\|_2^2.$$

In our simulations, the time interval and sampling rate are chosen so that $R_{x_i,x_j}(\tau)$ gives a good approximation of $\tau^*_{i,j}$. For instance, for the HR system, we select the fast variable (x-coordinate), so the time series has a sufficiently large number of cycles for the chosen time interval and step size.

For the case of phase synchronization (where only phase difference is relevant), the $\tau^*_{i,j}$ for every $i \neq j$ encodes all information about different final states, that is, they distinguish between chimera, complete synchronous, and completely incoherent states. In the generalized version of synchronization discussed here, not only are the $\tau^*_{i,j}$ important but so is the difference between the 'profile' of the time series, so $L(i,j,\tau^*_{i,j})$ should be taken into consideration as well. Therefore the state of the pair (i,j) can be further characterized by the ordered pair $(\tau^*_{i,j}, L(i,j,\tau^*_{i,j}))$.

We are now ready to define the Vector Pattern State (VPS) for all $(i \neq j)$

$$e^l = (\tau^{*,l}_{1,2}, \tau^{*,l}_{1,3}, \dots, \tau^{*,l}_{N-1,N}, \beta L^l(1,2,\tau^{*,l}_{1,2}), \beta L^l(1,3,\tau^{*,l}_{1,3}), \dots, \beta L^l(N-1,N,\tau^{*,l}_{N-1,N}), \tag{12}$$

where $\beta > 0$ allows for scaling the relative importance of the phase shift vs. the similarity measure between the time series. Each initial condition will lead to its own VPS. Though such states may not be unique, for instance, an initial condition that leads to CS will have the VPS: $e^l = (0,0,0,...,0)$ (in the limit as $t \to \infty$), and thus each initial condition that leads to CS will have the same VPS. In the above mentioned case of phase synchronization, the VPS will be given by $e^l = (\tau^*_{1,2}, \tau^*_{1,3}, \dots, \tau^*_{N-1,N}, 0, 0, \dots, 0)$ (in the limit as $t \to \infty$).

4.2 Mapping the Basin Structure

In the last section, we described our VPS so as to compare components of network coupled dynamics. With this, we can map the entire space of initial conditions to decide on a basin structure. The VPS allows for the characterization of states that may have both a phase shift as well as differences in the 'shape' of the time series. As discussed above, in the CS scenario, all components of the VPS will be ≈ 0. Similarly in the phase synchronization, all components corresponding to $L(i, j, \tau^*_{i,j})$ will be 0, while the $\tau^*_{i,j}$ themselves become bounded from above. However, the utility of the VPS is best expressed in chimera states and generalized synchronization. When some of the vertices become asynchronous and others follow a CS state, the VPS will have some components nearly 0 and others large.

We examine the CS case in more detail, noting it is analogous to the more generalized synchronization. Finding the basin structure is straightforward if the pairwise distances (under whatever measure is chosen) between all oscillators are small enough. The system may be considered in CS and the initial condition leading to this state may be marked as one color. Any initial condition not leading to CS will then be assigned a different color. However, this becomes much more challenging in chimera states, since not all pairs will reach CS. It may seem that classifying all states that have at least one synchronized pair as well as at least one desynchronized pair in the same basin will be sufficient, but chimera states range from being nearly incoherent to being nearly coherent, and classifying all as the same may hide very rich structure.

To resolve this, each VPS (e^l) is stacked into a matrix,

$$E = \begin{bmatrix} e^1 \\ e^2 \\ \vdots \\ e^S \end{bmatrix}, \tag{13}$$

where S is the number of VPSs or sample size. This matrix encodes all final states evolving from each initial condition, which are indexed by l. E is then clustered, in this case using k-means (see Section 4.3), with each cluster assigned a color in the basin plot. This leaves the choice of k, which was done using a variation on the elbow method. An example of how this is done is shown in Fig. 5.

In the elbow method, after a cluster has been chosen from k-means, the value of $W(C)$ from Eq. 16 becomes the dependent variable, with k as the independent variable. When examining this relationship across multiple k's

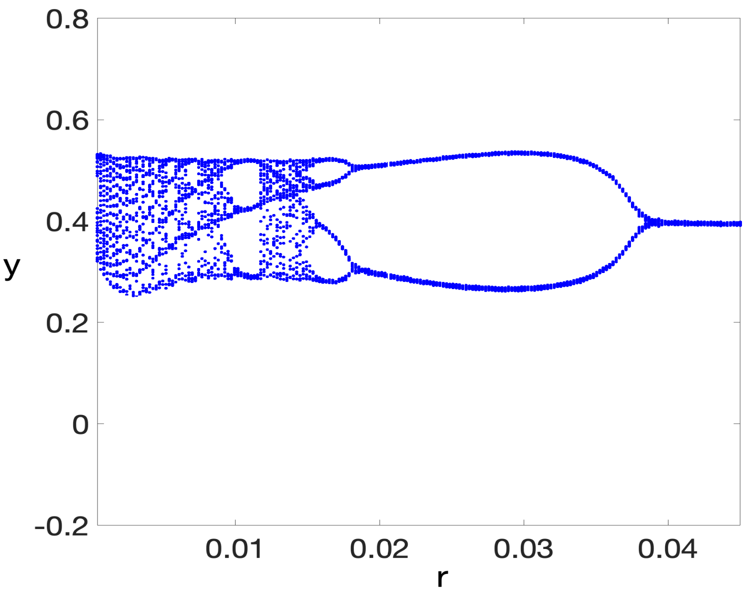

Supplementary Figure 4: The bifurcation diagram for an isolated HR oscillator. The parameters are $a = 1, b = 3, c = 1, d = 5, x_R = -0.5(1 + \sqrt{5})$, $s = 4$, and $I = 3.27$ [11].

generally there will be a discontinuity that is clearly visible, which will be the optimal value, though there are situations where this can fail [16]. In a slight variation, to identify the elbow, a log-log plot is used instead, and the last k before the majority of values fall on the linear trend is selected as the 'elbow' as shown in Fig. 5 (b).

The effects varying k has on the basin structure are shown in Fig. 6, the initial conditions chosen from the random plane shown here were drawn from a $1,500 \times 1,500$ grid, sampled over the unit square ($[0, 1]^2$). The initial conditions for all other oscillators were initially chosen uniformly at random from the uniform distribution $\mathcal{U}(-1, 1)$ and were then held constant with the exception of the initial conditions drawn from the random plane (which were sampled as discussed above). The complexity of the basin can be seen even for small values of k, the optimal k balances between too little and too much detail in the basin structure. In Fig. 6, $k = 8$ was found to be optimal using the version of the elbow method discussed above. Note that the basin used in this example differs from the one shown in Fig. 5, hence the different values of k.

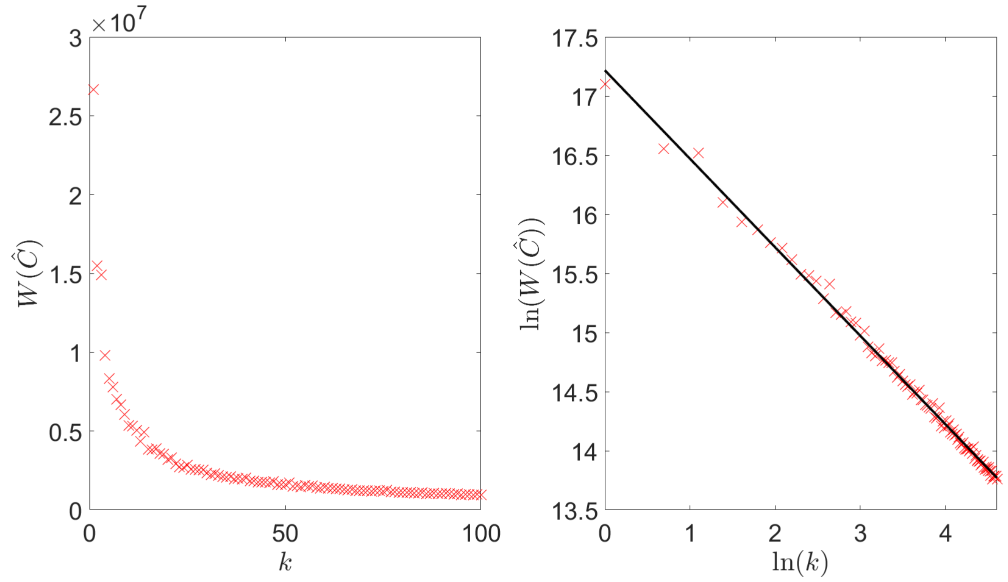

Supplementary Figure 5: The elbow method. (a) The traditional version of the elbow method relies on examining the plot of $W(\hat{C})$ and finding a point discontinuity, which is then chosen as k. Here it is challenging to find a clear elbow. (b) Instead, $\ln(k)$ vs. $\ln(W(\hat{C}))$ is plotted, with a linear fit determined. The best k value is chosen to be the last value before which the majority of the points fall on the line of best fit, in this example $k = 5$.

4.3 Clustering and k-Means

Often insights into a problem can be drawn by clustering data together to infer meaningful relationships. Numerous methods exist to perform clustering, however, k-means is a popular, robust, and relatively computationally inexpensive method for clustering a 'cloud' of data points together. With data $x \in \mathbb{R}^{S \times d}$, where S is the sample size and d is the dimension, and making the assumption that data is all of the quantitative types so that distance has meaning, then k-means is performed by minimizing the within cluster mean squared Euclidean distance between points. Choosing $1 \leq k \leq S$, let C be a clustering $C = \{C_1, C_2, ..., C_k\}$, with $C_k \subset \{1, 2, ..., S\}$. Then we define the dissimilarity measure d to be the squared Euclidean distance [16]:

$$d(x, y) = ||x - y||_2^2, \tag{14}$$

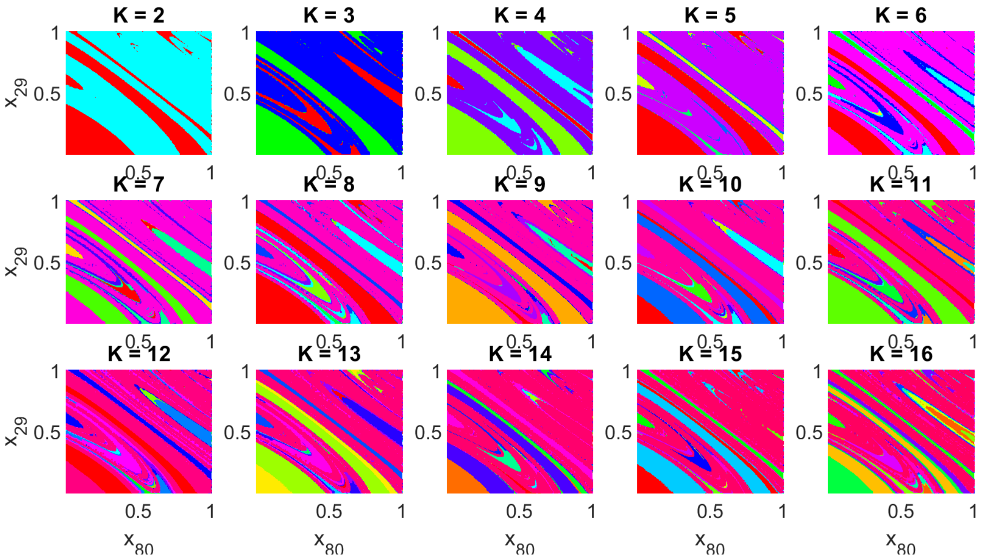

Supplementary Figure 6: Effect of varying k on the basin plots. Even for small k, a complex basin structure is observed. The choice of k can be seen as a trade off between too much and too little detail in this context.

and the within cluster mean

$$\bar{x}_k = \frac{1}{N_k} \sum_{i=1}^{S} I(i \in C_k) \cdot x_i, \tag{15}$$

where $N_k = \sum_{i=1}^{S} I(i \in C_k)$ and I is the indicator function. The within cluster squared error (W) is defined as,

$$W(C) = \sum_{j=1}^{k} \sum_{i=1}^{S} I(i \in C_j) d(x_i, \bar{x}_j). \tag{16}$$

Note that $d(x_i, \bar{x}_j)$ is the distance between a data point and the centroid of the cluster it is in. The k-means problem is to minimize this within cluster squared error, that is to find the cluster $\hat{C}$ with

$$W_k(\hat{C}) = \min_C W(C). \tag{17}$$

As noted above, there are numerous clustering techniques, and one can even generalize k-means by designing the distance function $d(x, y)$ to be a different dissimilarity measure than the more common squared Euclidean distance, such a clustering method is called k-medoids [16].

5 Networks of coupled oscillators

Here, we show that the construction of basins of attractions via VPS can also be applied to networks of coupled oscillators. First, we give details about the network dynamics used in the main text. Subsequently, we illustrate our approach to other network dynamics: Kuramoto oscillators and Hénon maps. In all cases our approach is able to capture the basin structure, illustrating its applicability.

5.1 Coupled HR neurons

Hindmarsh and Rose (HR) developed a model of neuronal firing in the brain [17]. We have used this model as a complex network of coupled oscillators as a semi-synthetic system using a true DTI network. Each HR neuron follows the following dynamics:

$$f(\mathbf{x}) = \begin{bmatrix} y - ax^3 + bx^2 - z + I \\ c - dx^2 - y \\ r[s(x - x_R) - z], \end{bmatrix} \tag{18}$$

where $\mathbf{x} = [x, y, z]^T$. The parameters a, b, c and d are all polynomial parameters, generally they are chosen to be $a = 1, b = 3, c = 1, d = 5$ following [17] and in this work we do the same. Varying b allows for switching between bursting and spiking behavior, I mimics the membrane input potential, r controls how quickly the slow variable (z) varies, s governs adaptation, and x_R represents the resting potential. Additionally, two different coupling functions were used, the first one,

$$h_1(\mathbf{x}_i, \mathbf{x}_j) = \begin{bmatrix} x_j - x_i \\ y_j - y_i \\ z_j - z_i \end{bmatrix}, \tag{19}$$

and the other one with

$$h_2(\mathbf{x}_i, \mathbf{x}_j) = \begin{bmatrix} 0 \\ y_j - y_i \\ 0 \end{bmatrix} - \alpha(x_i - V_{syn}) \begin{bmatrix} [1 + e^{-\lambda(x_j - \theta_{syn}}]^{-1} \\ 0 \\ 0 \end{bmatrix} \tag{20}$$

For the standard coupling scheme with h_1 given in Eq. 19, the values of a, b, c, d, x_R, and s are selected based on earlier research works, see [11, 17]. In this work, we set these parameters to $x_R = -0.5(1+\sqrt{5})$, $s = 4$, and $I = 3.27$. This will be referred to as the standard HR model. The value of x_R is chosen from the condition $\dot{x}_i = \dot{y}_i = 0$ while z_i (the slow variable) is neglected. With

this choice of parameters, and depending on r, an isolated HR neuron can exhibit a periodic time evolution. To decide if the time evolution is periodic or not, we follow the approach of Poincare [43]. Fig. 4 highlights how the behavior of the system changes when the parameter r is varied. For a given set of parameters, the number of cuts a moving HR neuron makes on the Poincaré plane is counted. The Poincaré plane is any plane transverse to a time-continuous flow, which in this case is the $y-z$ plane located at $x=0$. Fixing I and decreasing r towards 0, the y coordinates of all intersections on the Poincare plane are shown, such that $x(t) < 0$ and $x(t+dt) > 0$, where dt denotes the integration time step. The resulting plot (Fig. 4) is called a bifurcation diagram and the type of bifurcation is a period-doubling bifurcation of limit cycles. A finite number of cuts (let's say N_c) indicate a periodic trajectory with period N_c, while the limit $N_c \to \infty$ shows a chaotic evolution.

An alternative model, which includes chemical coupling in addition to the electrical coupling, that is with h_2 from Eq. 20, the parameters are: $a=1, b=3, c=1, d=5, s=4, r=0.005, x_R=-1.6, I=3.25, \sigma=0.5, \alpha=0.03, V_{syn}=2, \theta_{syn}=-0.25$ and $\lambda=10$, which is known to contain chimeras [18]. Here the chemical and electrical adjacency matrices are assumed to be the same, unlike in [18]. This will be referred to as the chemical coupling model. An example of the dynamics is shown in Fig. 2 (c) and (d), and a version of this model where the parameter a for each oscillator is drawn from a normal distribution is shown in Fig. 2 (e) and (f).

5.2 Two populations of globally coupled oscillators

Here, we consider the case of two small size populations that are globally coupled to each other [1]. We consider two coupled populations as illustrated in Figure 7 a)

$$\dot{\theta}_i^k = \omega + \sum_{k'=1}^{2} \frac{\sigma_{kk'}}{N_{k'}} \sum_{j=1}^{N_{k'}} \sin(\theta_j^{k'} - \theta_i^k - \alpha), \quad i = 1, \dots, N_k, \tag{21}$$

where θ_i^k is the phase of the i-th oscillator in population $k \in \{1,2\}$ and ω is the oscillator frequency. Based on earlier works [1, 24, 31], we suppose that $\sigma_{11} = \sigma_{22} = \mu > 0$, and $\sigma_{12} = \sigma_{21} = \nu > 0$, with $\mu > \nu$. Also, by rescaling time, we may set $\mu + \nu = 1$ and introduce useful parameters $A = \mu - \nu$ and $\gamma = \pi/2 - \alpha$ that determine the existence of chimeras states in the system. For identical oscillators, we can change coordinates to the rotating frame ω, $\theta_i^k \mapsto \theta_i^k - \omega t$, so the natural frequency can be fixed at $\omega = 0$.

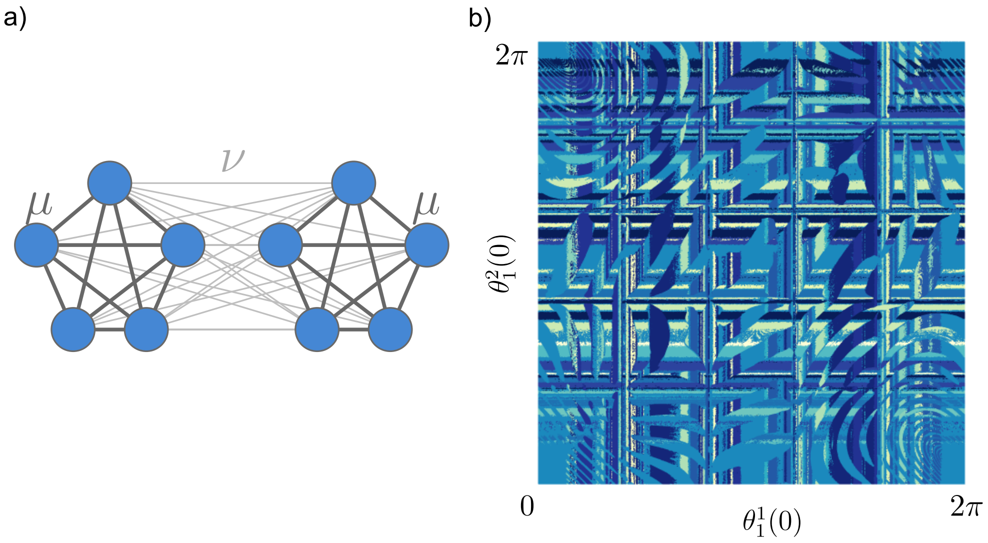

Supplementary Figure 7: Basin structure of two globally coupled populations. (a) Two globally coupled populations of five nodes. The (intra-)coupling strength inside a population and (inter-)coupling strength between populations are given by μ and ν, respectively, with $\mu > \nu$. (b) Two-dimensional section of the state space showing basins of the 18 (clustered) distinct states identified by the VPS of the two globally coupled populations. There is a symmetry among the basins with respect to reflections across the diagonals, which originates from the reflection symmetry of the network. To construct the VPS, we use $\beta = 1$ in Equation (12) and a grid of $1,257 \times 1,257$ uniformly sampled initial conditions.

The system (21) has full permutation symmetry, allowing a low-dimensional description in terms of macroscopic variables in the thermodynamic limit ($N_k \to \infty$) [1]. In particular, the authors in [24] use the resulting reduced system to describe the basins of attraction of chimera states. The basin structure corresponds to the infinite size system, but it is expected to remain roughly the same for large population sizes [31]. We tested our VPS construction (results not shown), reproducing a basin structure that contains a few distinct states, as predicted by the infinite size system [24]. Here, we consider the interesting case when the population size is small, and the dynamics of the full system deviate from the evolution of the reduced system [31]. In fact, in this scenario, the evolution still has a low-dimensional description but the macroscopic variables do not evolve in a closed-form equation, so the reduced system becomes intractable analytically, see [25] for details.

We consider the case of $N = 5$, $A = 0.1$ and $\gamma = 0.025$, where it is known that stable chimeras exist [1, 24, 31]. For initial conditions we consider equally spaced phases over the circle, i.e., for a fixed $\vartheta \in [0, 2\pi]$

$$\theta_i^1(0) = \frac{2\pi i}{N}, \quad i = 1, \dots, N_1,$$
$$\theta_i^2(0) = \frac{2\pi i}{N} + \vartheta, \quad i = 1, \dots, N_2,$$

so the system starts close to an incoherence state.

We consider $\vartheta = 0.1$, transient time of $2,000$ time steps, and integrate over 1000 more time steps to obtain the final state, sampling every 0.02 time step. The basin plot is performed in the plane $(\theta_1^1(0), \theta_1^2(0))$ for which the initial conditions are varied over the interval $[0, 2\pi]$, see Figure 7 (b). We see that the basin has a coexistence of different chimera states and (complete) phase synchronization states, as described in [31], and our method is capable of identifying these cases.

5.3 Coupled oscillators in networks

Here, we apply the VPS construction to networks that are not globally coupled, but rather have some missing links. In this scenario, instead of the network allowing all permutation symmetries, only a few permutations are possible. We consider the following equations of motion for the oscillators

$$\dot{\theta}_i = \sigma \sum_{j=1}^{N} [A]_{i,j} \sin(\theta_j - \theta_i - \alpha), \quad i = 1, \dots, N, \tag{22}$$

where $\sigma = 0.01$ is the overall coupling strength. The adjacency matrix A represents a network that does not have full permutation symmetry. To generate this network we initiate the globally coupled network, as depicted in Figure 7 (a), and remove uniformly at random one edge from the graph. The resulting graph has two new clusters that are depicted in different colors, which is calculated via Sage [41]. See Figure 8 for an illustration of the (random) network used in our numerical results.

We consider a time interval of $5,000$ time steps and sample the trajectory at every 0.02 time step. Also, the parameters are $\sigma = 0.01$ and as before $\alpha = \pi/2 - \gamma$ with $\gamma = 0.025$. Choosing the same two-dimensional section as previously, we construct the basin structure via VPS, as illustrated in Figure 8 (b), using five clusters that are determined by the elbow method discussed earlier. Here, we consider a transient time of $4,000$ time steps and calculate

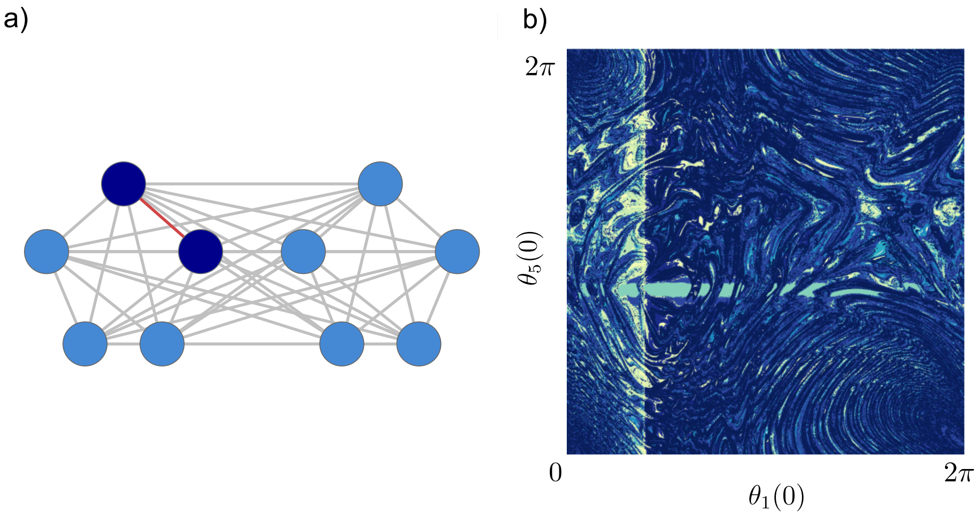

Supplementary Figure 8: (a) Network with one single link removed, breaking the full permutation symmetry of the globally coupled network. Nodes with different colors correspond to the new clusters formed from the permutation symmetry group. The red edge corresponds to a randomly selected edge that was removed from the globally coupled network. (b) Two-dimensional section of the state space showing basins of 12 (clustered) distinct states. To construct the VPS, we use $\beta = 1$ in Equation (12) and a grid with $1,257 \times 1,257$ uniformly sampled initial conditions.

the similarity measure and cross-correlation, over the remaining $1,000$ time steps.

We also test the performance of the method for another network structure, where two links are removed, see Figure 9 a). We consider a time interval of $15,000$ time steps, discarding the first $14,000$ time steps as transient, and sample the trajectory at every 0.05 time step. The parameters are $\alpha = \pi/2 - \gamma$ with $\gamma = 0.075$ and $\sigma = 0.01$. Figure 9 b) displays the basin structure identified by our approach, where a zoom region has been enlarged, confirming that the basin is intermingled.

In both cases, the basins show that the systems have a high sensitivity to initial conditions, so our method is able to characterize such basins that have intermingled structures. It remains an interesting line of research to describe the mechanism behind the chaotic dynamics since it differs from the globally coupled scenario where a low-dimensional description is available [8].

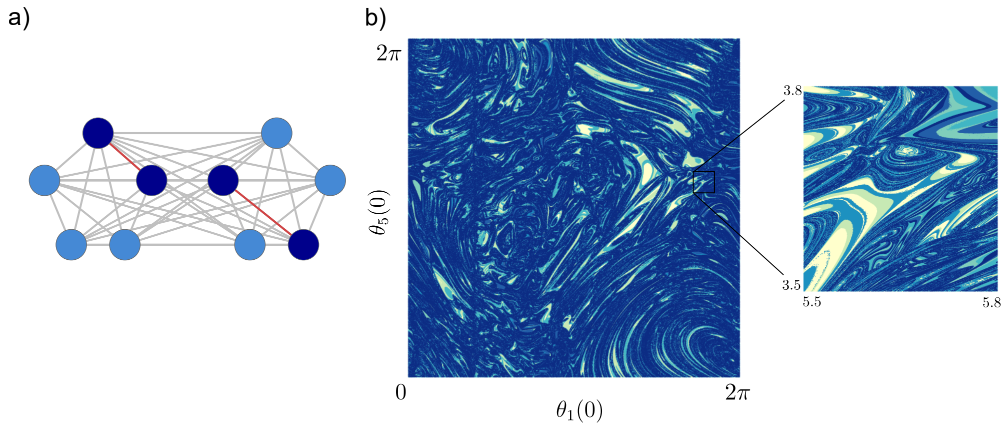

Supplementary Figure 9: (a) Network with two links removed, breaking the full permutation symmetry of the globally coupled network. Nodes with different colors correspond to the new clusters formed from the permutation symmetry group. The red edges correspond to randomly selected edges that were removed from the globally coupled network. (b) Two-dimensional section of the state space showing basins of 7 (clustered) distinct states. To construct the VPS, we use $\beta = 1$ in Equation (12) and a grid with $1,257 \times 1,257$ and 624×624 for the left and right panel, respectively, uniformly sampled initial conditions. The parameters are $\gamma = 0.075$ and $\sigma = 0.01$.

5.4 VPS for phase oscillators

Different from the HR system, when we consider coupled oscillators, the time series is given by phases $\{\theta_i(t)\}_{t\geq 0}$ in the circle. To construct the VPS, we perform an embedding into $\mathbb{R}$, so we can use the same similarity measure introduced in Section 4.1. More precisely, for each oscillator time series, we consider

$$x_i(t) = \cos(\theta_i(t)), \quad i = 1, \ldots, N.$$

5.5 Coupled Hénon maps

To allow the system to settle on to the attractor we use $20,000$ time steps. The final $1,000$ time steps are utilized for the construction of the VPS. Following several of the above examples, we use only the x-component for the VPS. The basin shown contains $1,000,000$ initial conditions sampled in a grid of $[-3,3]^2$ for the x and y component of a randomly chosen node. The initial condition for all other nodes is set to $(0,0)^T$. Though we are using a different network structure, we find a very similar basin structure to that

of [36]. This basin appears to be riddled, which indicates how general this phenomenon is. Additionally, it is clear that non-trivial symmetry is not necessary for riddled basins.

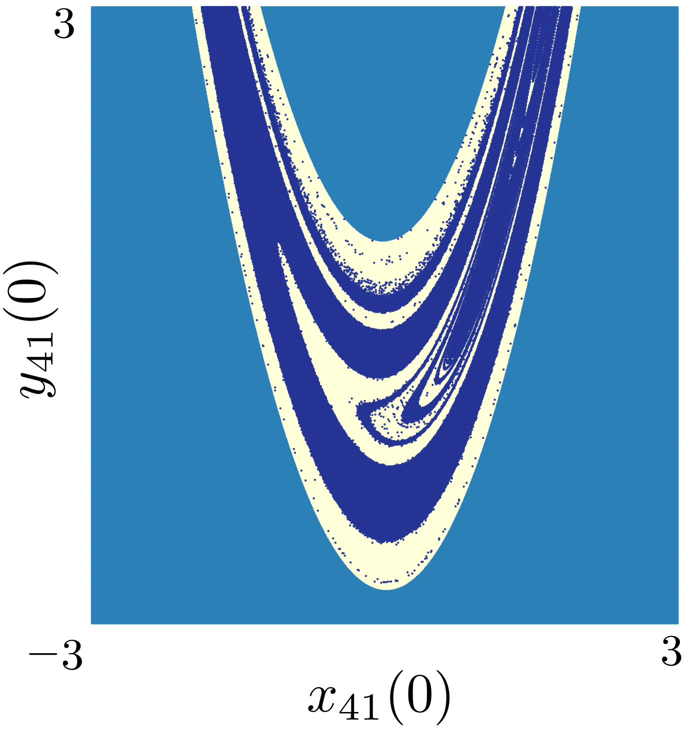

Supplementary Figure 10: Basin structure in the Hénon system for a randomly chosen x,y plane. The network is the same DTI network shown in Fig.1 in the main text. This structure appears to have a riddled basin.

References

[1] Daniel M. Abrams, Rennie Mirollo, Steven H. Strogatz, and Daniel A. Wiley. Solvable model for chimera states of coupled oscillators. Phys. Rev. Lett., 101:084103, Aug 2008.

[2] Daniel M. Abrams and Steven H. Strogatz. Chimera States for Coupled Oscillators. Phys. Rev. Lett., 93:174102, Oct 2004.

[3] JC Alexander, James A Yorke, Zhiping You, and Ittai Kan. Riddled basins. International Journal of Bifurcation and Chaos, 2(04):795–813, 1992.

[4] Michael F Barnsley. Fractals everywhere. Academic press, 2014.

[5] Michael F Barnsley and Stephen Demko. Iterated function systems and the global construction of fractals. Proceedings of the Royal Society of London. A. Mathematical and Physical Sciences, 399(1817):243–275, 1985.

[6] Vladimir N Belykh, Igor V Belykh, and Erik Mosekilde. Cluster synchronization modes in an ensemble of coupled chaotic oscillators. Physical Review E, 63(3):036216, 2001.

[7] Vladimir N Belykh, Grigory V Osipov, Valentin S Petrov, Johan AK Suykens, and Joos Vandewalle. Cluster synchronization in oscillatory networks. Chaos: An Interdisciplinary Journal of Nonlinear Science, 18(3):037106, 2008.

[8] Christian Bick, Mark J. Panaggio, and Erik A. Martens. Chaos in Kuramoto oscillator networks. Chaos: An Interdisciplinary Journal of Nonlinear Science, 28(7), 07 2018. 071102.

[9] Leonardo Bonilha, Ezequiel Gleichgerrcht, Julius Fridriksson, Chris Rorden, Jesse L. Breedlove, Travis Nesland, Walter Paulus, Gunther Helms, and Niels K. Focke. Reproducibility of the Structural Brain Connectome Derived from Diffusion Tensor Imaging. PLOS ONE, 10(9):1–17, 09 2015.

[10] Bernard Cazelles. Dynamics with riddled basins of attraction in models of interacting populations. Chaos, Solitons & Fractals, 12(2):301–311, 2001.

[11] S. R. Dtchetgnia Djeundam, R. Yamapi, T. C. Kofane, and M. A. Aziz-Alaoui. Deterministic and stochastic bifurcations in the Hindmarsh-Rose neuronal model. Chaos: An Interdisciplinary Journal of Nonlinear Science, 23(3):033125, 2013.

[12] K. J Falconer. Fractal geometry mathematical foundations and applications. Wiley, Chichester New York, 1990.

[13] Jeremie Fish, Alexander DeWitt, Abd AlRahman R. AlMomani, Paul J. Laurienti, and Erik Bollt. Entropic regression with neurologically motivated applications. Chaos: An Interdisciplinary Journal of Nonlinear Science, 31(11):113105, 2021.

[14] Celso Grebogi, Edward Ott, and James A Yorke. Fractal basin boundaries, long-lived chaotic transients, and unstable-unstable pair bifurcation. Physical Review Letters, 50(13):935, 1983.

[15] HS Greenside, A Wolf, J Swift, and T Pignataro. Impracticality of a box-counting algorithm for calculating the dimensionality of strange attractors. Physical Review A, 25(6):3453, 1982.

[16] Trevor Hastie, Robert Tibshirani, and Jerome Friedman. Elements of Statistical Learning: Data Mining, Inference, and Prediction. Springer series in statistics. Springer, New York, 2009.

[17] J. L. Hindmarsh, R. M. Rose, and Andrew Fielding Huxley. A model of neuronal bursting using three coupled first order differential equations. Proceedings of the Royal Society of London. Series B. Biological Sciences, 221(1222):87–102, 1984.

[18] Johanne Hizanidis, Nikos E Kouvaris, Gorka Zamora-López, Albert Díaz-Guilera, and Chris G Antonopoulos. Chimera-like states in modular neural networks. Scientific reports, 6(1):1–11, 2016.

[19] Kunihiko Kaneko. Clustering, coding, switching, hierarchical ordering, and control in a network of chaotic elements. Physica D: Nonlinear Phenomena, 41(2):137–172, 1990.

[20] Yoshiki Kuramoto and Dorjsuren Battogtokh. Coexistence of coherence and incoherence in nonlocally coupled phase oscillators. arXiv preprint cond-mat/0210694, 2002.

[21] Carlo R Laing. Chimeras in networks of planar oscillators. Physical Review E, 81(6):066221, 2010.

[22] Soumen Majhi, Bidesh K Bera, Dibakar Ghosh, and Matjaž Perc. Chimera states in neuronal networks: a review. Physics of life reviews, 28:100–121, 2019.

[23] Benoit B Mandelbrot. The Fractal Geometry of Nature. W. H. Freeman, New York, 1983.

[24] Erik A Martens, Mark J Panaggio, and Daniel M Abrams. Basins of attraction for chimera states. New Journal of Physics, 18(2):022002, feb 2016.

[25] Seth A. Marvel, Renato E. Mirollo, and Steven H. Strogatz. Identical phase oscillators with global sinusoidal coupling evolve by Möbius group action. Chaos: An Interdisciplinary Journal of Nonlinear Science, 19(4), 10 2009. 043104.

[26] Steven W McDonald, Celso Grebogi, Edward Ott, and James A Yorke. Fractal basin boundaries. Physica D: Nonlinear Phenomena, 17(2):125–153, 1985.

[27] Iryna Omelchenko, Astero Provata, Johanne Hizanidis, Eckehard Schöll, and Philipp Hövel. Robustness of chimera states for coupled fitzhugh-nagumo oscillators. Physical Review E, 91(2):022917, 2015.

[28] Kenju Otsuka, Yoshinori Sato, and Jyh-Long Chern. Grouping of antiphase oscillations in modulated multimode lasers. Physical Review A, 54(5):4464, 1996.

[29] Edward Ott, JC Alexander, Ittai Kan, John C Sommerer, and James A Yorke. The transition to chaotic attractors with riddled basins. Physica D: Nonlinear Phenomena, 76(4):384–410, 1994.

[30] Edward Ott, John C Sommerer, James C Alexander, Ittai Kan, and James A Yorke. Scaling behavior of chaotic systems with riddled basins. Physical review letters, 71(25):4134, 1993.

[31] Mark J. Panaggio, Daniel M. Abrams, Peter Ashwin, and Carlo R. Laing. Chimera states in networks of phase oscillators: The case of two small populations. Phys. Rev. E, 93:012218, Jan 2016.

[32] L. M. Pecora, Francesco Sorrentino, Aaron M. Hagerstrom, Thomas E. Thomas E. Murphy, and Rajarshi Roy. Cluster synchronization and isolated desynchronization in complex networks with symmetries. Nature Communication, 5(4079):1–8, 2014.

[33] Louis M. Pecora and Thomas L. Carroll. Master stability functions for synchronized coupled systems. Phys. Rev. Lett., 80:2109–2112, Mar 1998.

[34] Arkady Pikovsky, Michael Rosenblum, and Jurgen Kurths. Synchronization: A Universal Concept in Nonlinear Sciences. Cambridge U. Press, New York, 2001.

[35] David A Russell, James D Hanson, and Edward Ott. Dimension of strange attractors. Physical Review Letters, 45(14):1175, 1980.

[36] Vagner dos Santos, JD Szezech Jr, Antonio Marcos Batista, Kelly C Iarosz, Murilo da Silva Baptista, Hai Peng Ren, Celso Grebogi, Ricardo Luiz Viana, Iberê Luiz Caldas, Yuri L Maistrenko, et al. Riddling: Chimera's dilemma. Chaos: An Interdisciplinary Journal of Nonlinear Science, 28(8):081105, 2018.

[37] Nirupam Sarkar and Bidyut Baran Chaudhuri. An efficient differential box-counting approach to compute fractal dimension of image. IEEE Transactions on systems, man, and cybernetics, 24(1):115–120, 1994.

[38] Gautam C. Sethia and Abhijit Sen. Chimera states: The existence criteria revisited. Phys. Rev. Lett., 112:144101, Apr 2014.

[39] Francesco Sorrentino and Louis Pecora. Approximate cluster synchronization in networks with symmetries and parameter mismatches. Chaos: An Interdisciplinary Journal of Nonlinear Science, 26(9):094823, 2016.

[40] Harry Eugene Stanley and Nicole Ostrowsky. On growth and form: fractal and non-fractal patterns in physics, volume 100. Springer Science & Business Media, 2012.

[41] William Stein and David Joyner. SAGE: Software for Algebra and Geometry Experimentation. https://www.sagemath.org, 2005. Accessed: 2023-05-04.

[42] Ian Stewart, Martin Golubitsky, and Marcus Pivato. Symmetry groupoids and patterns of synchrony in coupled cell networks. SIAM Journal on Applied Dynamical Systems, 2(4):609–646, 2003.

[43] Steven H Strogatz. Nonlinear dynamics and chaos with applications to physics, biology, chemistry, and engineering. Westview Press, 2015.

[44] Jie Sun, Erik M Bollt, and Takashi Nishikawa. Master stability functions for coupled nearly identical dynamical systems. EPL (Europhysics Letters), 85(6):60011, 2009.

[45] Ruopeng Wang, Thomas Benner, Alma Gregory Sorensen, and Van Jay Wedeen. Diffusion toolkit: a software package for diffusion imaging data processing and tractography. In Proc Intl Soc Mag Reson Med, volume 15. Berlin, 2007.

[46] Damián H Zanette and Alexander S Mikhailov. Condensation in globally coupled populations of chaotic dynamical systems. Physical Review E, 57(1):276, 1998.

[47] Yuanzhao Zhang and Sean P Cornelius. A catch-22 of reservoir computing. arXiv preprint arXiv:2210.10211, 2022.
